# Cell Cycle-Related Kinase (CCRK) regulates ciliogenesis and Hedgehog signaling in mice

**DOI:** 10.1371/journal.pgen.1006912

**Published:** 2017-08-17

**Authors:** Ashley Snouffer, Desmond Brown, Hankyu Lee, Jonathon Walsh, Floria Lupu, Ryan Norman, Karl Lechtreck, Hyuk Wan Ko, Jonathan Eggenschwiler

**Affiliations:** 1 Department of Genetics, University of Georgia, Athens, GA, United States of America; 2 Department of Molecular Biology, Princeton University, Princeton, NJ, United States of America; 3 College of Pharmacy, Dongguk University-Seoul, Goyang, Korea; 4 Department of Cellular Biology, University of Georgia, Athens, GA, United States of America; Emory University School of Medicine, UNITED STATES

## Abstract

The Hedgehog (Hh) signaling pathway plays a key role in cell fate specification, proliferation, and survival during mammalian development. Cells require a small organelle, the primary cilium, to respond properly to Hh signals and the key regulators of Hh signal transduction exhibit dynamic localization to this organelle when the pathway is activated. Here, we investigate the role of Cell Cycle Related kinase (CCRK) in regulation of cilium-dependent Hh signaling in the mouse. Mice mutant for *Ccrk* exhibit a variety of developmental defects indicative of inappropriate regulation of this pathway. Cell biological, biochemical and genetic analyses indicate that CCRK is required to control the Hedgehog pathway at the level or downstream of Smoothened and upstream of the Gli transcription factors, Gli2 and Gli3. *In vitro* experiments indicate that *Ccrk* mutant cells show a greater deficit in response to signaling over long time periods than over short ones. Similar to *Chlamydomonas* mutants lacking the CCRK homolog, LF2, mouse *Ccrk* mutant cells show defective regulation of ciliary length and morphology. *Ccrk* mutant cells exhibit defects in intraflagellar transport (the transport mechanism used to assemble cilia), as well as slowed kinetics of ciliary enrichment of key Hh pathway regulators. Collectively, the data suggest that CCRK positively regulates the kinetics by which ciliary proteins such as Smoothened and Gli2 are imported into the cilium, and that the efficiency of ciliary recruitment allows for potent responses to Hedgehog signaling over long time periods.

## Author summary

The importance of cilia in development and disease has become broadly appreciated in recent years due in part to their roles in signal transduction. Despite this attention, crucial aspects of ciliary assembly and function, such as the mechanisms controlling ciliary assembly and the signal transduction events occurring in cilia, remain unclear. Cilia play a central role in sensing and transducing Hedgehog signals in the context of mammalian embryogenesis and in a variety of cancers. Here, we investigate the functions of Cell Cycle Related Kinase (CCRK), which plays an evolutionarily conserved function in the assembly of cilia and flagella. We find that mouse CCRK positively and negatively regulates ciliary length. We show that CCRK controls multiple aspects of Hedgehog signaling *in vivo* and *in vitro* by regulating the processing and activities of the Gli transcription factors. Our data suggest that CCRK controls Hedgehog signaling by promoting the efficient ciliary import of core signaling components.

## Introduction

### The role of Hedgehog signaling in vertebrate development

In the mammalian embryo, the Hedgehog (Hh) signaling pathway controls cell proliferation, cell survival, and tissue patterning (cell fate specification and differentiation) in most tissues such as the developing nervous system, skeleton, skin, and internal organs (reviewed in [1]). The role of Hh signaling in tissue patterning has been most extensively studied in the context of the spinal neural tube. In this system, the Sonic Hedgehog (Shh) ligand functions as a morphogen [2]; cells experiencing the strong, intermediate, low, or absent signaling (in a ventral-to-dorsal order) adopt the floor plate, motor neuron, V0-V2 interneuron, or dorsal interneuron fates, respectively. The strength of signaling is determined by the amount of ligand cells experience, as well as the duration of that exposure [3].

### Mammalian Hedgehog signaling and the primary cilium

At the surface of signal responding cells, Hh ligands bind to a complex including the transmembrane receptor Patched (Ptch1). In the absence of the Hh ligand, Patched inhibits the seven-pass transmembrane signal transducer, Smoothened (Smo). Binding of Hh ligand to Patched relieves its inhibition on Smo, thereby activating it. In turn, active Smo regulates the activity of the Gli family transcription factors, through a process that is not fully understood.

Mammals have three *Gli* genes (*Gli1- 3*) [4]. *Gli1*, whose expression is strongly induced by Hh signaling, encodes a transcriptional activator of Hh targets but it is not required for embryonic Hh signal transduction. Gli2 and Gli3, however, are both necessary to control embryonic Hh signaling, functioning as activators or repressors of target genes. In the absence of Hh ligands, Gli2 and Gli3 are phosphorylated by a series of kinases, leading to proteasome-dependent processing into shorter repressor forms [5]. Unprocessed (full-length) forms are kept in a functionally inactive state due to their physical association with Suppressor of Fused (Sufu, [6]). Once Smo is activated by signaling, the phosphorylation pattern of Gli proteins is altered [7]). These changes prevent proteolytic processing (thus repressor formation) and they render the Gli proteins active such that they can enter the nucleus and activate the expression of a series of genes, including *Gli1* and *Ptch1*.

In mice, much of this signal transduction occurs in the primary cilium (reviewed in [8]). This relationship appears to be generally conserved among vertebrates including humans [9, 10]. The primary cilium, present on nearly every cell type in the body, is a microtubule-based appendage surrounded by an extension of the plasma membrane and anchored by the mother centriole [11]. It is assembled primarily through a mechanism termed Intraflagellar Transport (IFT, reviewed in [11]). IFT particles associate with ciliary cargo and microtubule motors, forming trains that are transported in an anterograde direction towards microtubule plus-ends (at the distal end of the cilium) by Kinesin II. IFT machinery and cargo to be exported from the cilium are loaded onto IFT particles that are returned to the cell body (retrograde transport towards microtubule minus-ends) through cytoplasmic Dynein 1b.

A large number of mutations have been identified in mice that block the formation of cilia or alter their assembly and all of these perturbations lead to changes (increase or decrease) in the activity of Hh signaling in the embryo [12–16]. Key regulatory components of the Hh pathway show dynamic changes in their localization to cilia, depending on the presence or absence of signals [17, 18]. In the absence of signals, Patched1 localizes to the cilium, whereas Smo shows little or no localization. Only a small amount of Gli2, Gli3, and Sufu localize to the cilium (where they concentrate at its tip). In the presence of signals, ciliary localization of Patched is lost, while Smo, Gli2, Gli3, and Sufu become enriched in the structure [17, 18]. A number of experiments indicate that this change in ciliary localization is important for the factors to execute signal transduction [19, 20]. Analysis of mouse mutants has shown that proper proteolytic processing of full-length Gli proteins to generate repressor forms is dependent on primary cilia [21]. In parallel, the ability of full-length Gli proteins to undergo differential phosphorylation, dissociate from Sufu, and activate Hh target genes also requires primary cilia [22]. Because of this, defects in ciliogenesis result in ligand-independent up-regulation of Hh targets, down-regulation or absence of ligand-dependent Hh responses, or a combination of the two effects.

### Regulation of ciliary assembly by a kinase, CCRK, and its homologs

Studies of a mutation that perturbs ciliogenesis and Hedgehog signaling in the mouse, *broadminded (bromi)*, implicated Cell Cycle-Related Kinase (CCRK, also known as Cdk20) in these processes [23].

CCRK shares homology with members of the Cyclin-dependent Kinase (CDK) family and it was originally identified in vertebrates as a Cdk2-activating protein [24]. However, subsequent work showed that CCRK has only a minor role in cell cycle progression [25], leaving the role of mammalian CCRK unclear.

CCRK homologs have been identified and studied in several species (*Chlamydomonas reinhardtii*, *C*. *elegans*, *Leishmania mexicana*, and *Danio rerio)* and, in all cases, disruption of the gene leads to defects in ciliary length and/or structure [23, 26–28]. The homolog in the algae *Chlamydomonas* is called Long Flagella 2 (LF2). The originally identified mutations in *lf2* cause flagella to be significantly longer than normal [26]. However, these mutations were subsequently found to be hypomorphic. In contrast, cells harboring a null allele, *lf2-6*, generate flagella with a broad range of lengths (either too long, too short, or flagella of different lengths on the same cell, [29]). Partial knockdown of *Ccrk* expression in cultured mammalian cells results in ciliary lengthening [30], as in *lf2* hypomorphic *Chlamydomonas* mutants [26], but the effect of complete loss of CCRK on Hedgehog signaling and ciliogenesis in mammals has not been previously determined.

Here, we find that the role of CCRK in mice is complex, as it positively and negatively controls both ciliary length and the activity of the Hh pathway. The results indicate that CCRK positively regulates import of ciliary cargo including Hh signaling components. They also suggest that efficient flux of Hh signaling components into, and out of, the cilium, is limiting for long-term, but not initial, Hh responses.

## Results

### Mouse CCRK is required for embryonic viability and neural patterning

**Fig 1:**
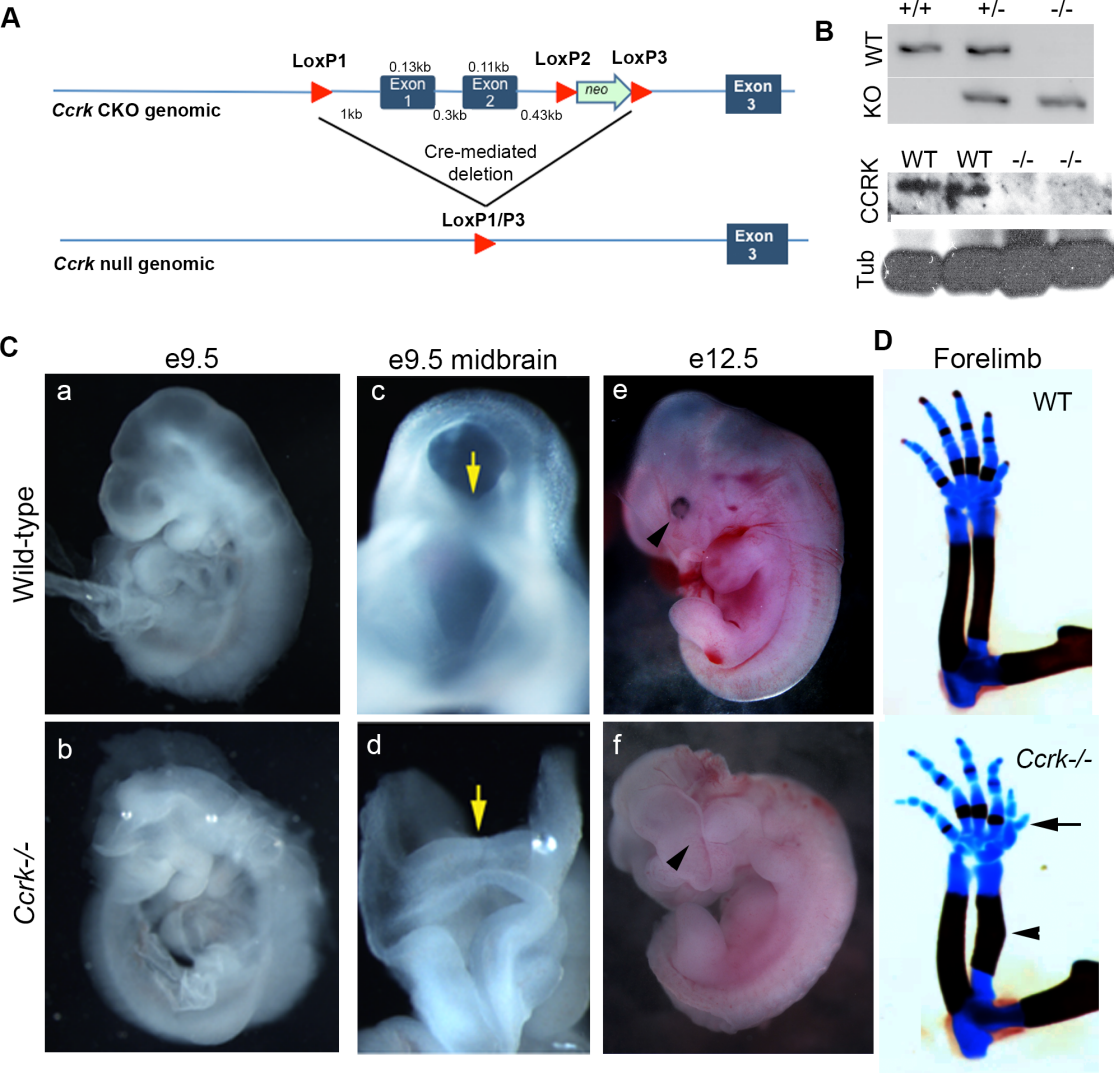
*Ccrk* mutants show pleiotropic embryonic phenotypes. (A) The targeting strategy for generation of a *Ccrk* null allele. Cre-mediated recombination was used to generate the null allele (*Ccrk^KO^*) from the floxed allele. (B) PCR confirmed mutagenesis and western blotting indicated that *Ccrk*^*KO*^ is a null allele. (C) Morphological features of embryonic day (E) 9.5 (b,d) and 12.5 (f) *Ccrk* mutants, such as exencephaly. Yellow arrows in c,d indicate the lack of the midbrain furrow in *Ccrk* mutants (d); black arrowheads in e,f highlight the eye defects in *Ccrk* mutants (f). (D) Limb skeletal defects, such as preaxial polydactyly (arrow) and achondroplasia of the radius and ulna (arrowhead).

We generated a mutation in the mouse *Ccrk* gene through gene targeting by removing the first two exons of the *Ccrk* genomic locus (see Materials and Methods). This allele, *Ccrk*^*KO*^, should be null, as it lacks promoter sequences, transcriptional and translational start sites, as well as the ATP binding site required for kinase activity **(Fig 1A)**. Western blotting of *Ccrk*^*KO*^ homozygous mutant embryonic extracts indicated that *Ccrk*^*KO*^ represents a null allele (**Fig 1B**).

*Ccrk*^*KO/KO*^ (hereafter referred to as *Ccrk*^*-/-*^) mutant embryos exhibited an embryonic phenotype closely resembling that of *bromi* mutant embryos (**Figs 1C and S1),** whereas *Ccrk*^*+/-*^ mice showed no apparent phenotype. *Ccrk*^*-/-*^ embryos survived until late gestation (embryonic day 17.5, E17.5). Like *broadminded* mutants, *Ccrk*^*-/-*^ mutant embryos exhibited a constellation of defects including exencephaly, mild preaxial polydactyly/limb skeletal defects, reduction of the retinal pigmented epithelium, and cleft palate (**Figs 1C, 1D and S1)**. We did not detect a noticeable difference in size between homozygotes and their wild-type littermates. In addition to failing to close the neural tube, *Ccrk*^*-/-*^ mutants lacked the midline furrow in the midbrain, suggesting that the most ventral, Shh-dependent, floor plate cells were not specified in such mutants. At neural patterning stages (E10.5) we found uniform low-level expression of *Ccrk* throughout the embryo and, as expected due to the mutation induced, *Ccrk*^*-/-*^ mutant embryos failed to show detectable expression (**S2 Fig**). The phenotypic features we observed have been described in other mouse mutants with defective Sonic Hedgehog signaling [31–33] and they are consistent with a role for CCRK in this pathway.

### CCRK is required in the spinal neural tube for responses to high levels of Shh and for repression of weak Hh pathway activity

**Fig 2:**
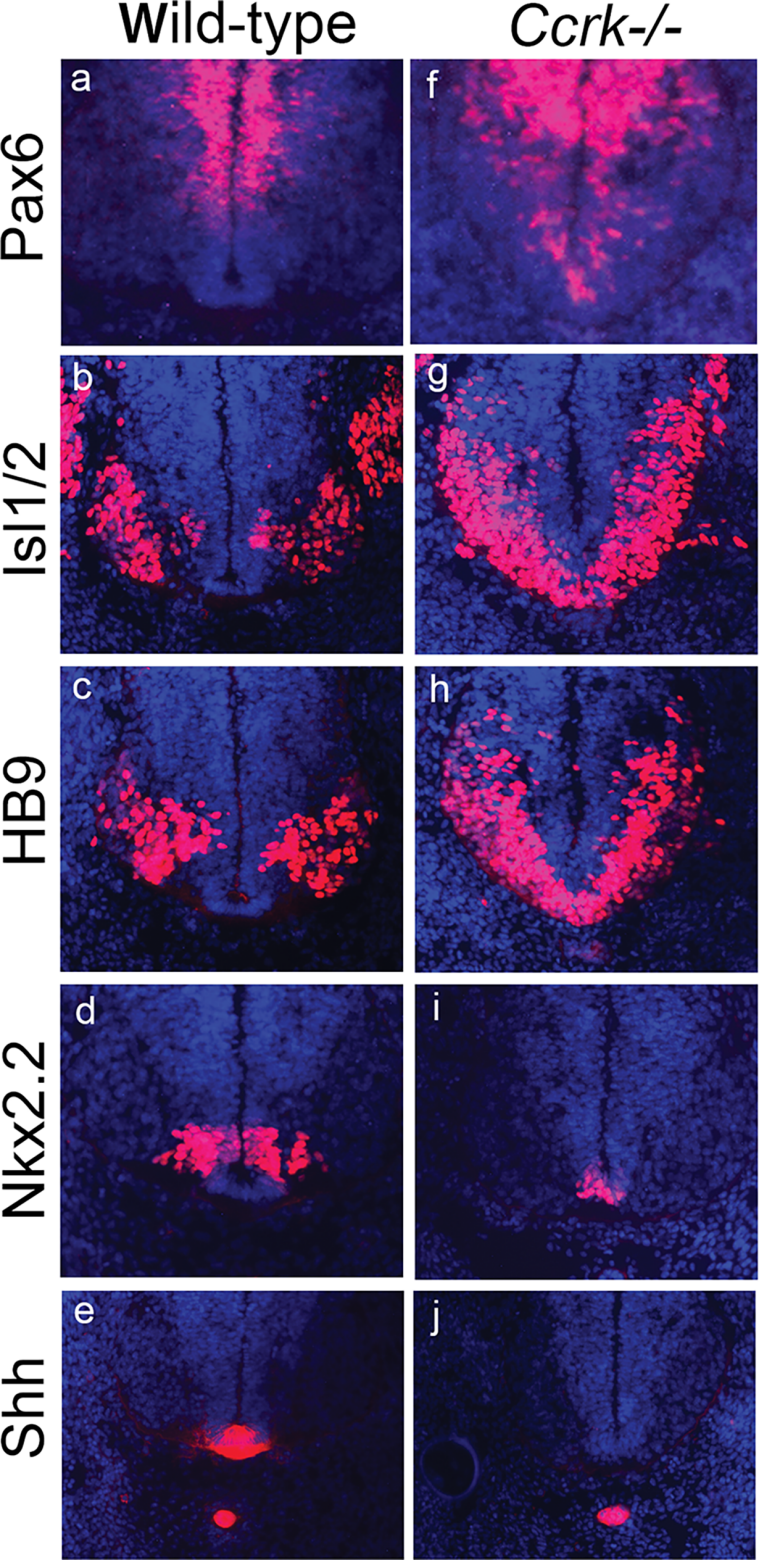
*Ccrk* mutants show defective Shh-dependent patterning of the neural tube. Sections through the brachial spinal neural tubes of E11.5 wild-type (a-e) and *Ccrk* mutants (f-j). Note in the mutant that the expression domain of Pax6 expands ventrally (f), the Isl1/2+ (g) and HB9+ (h) motor neuron domains expand both ventrally and dorsally, the Nkx2.2 domain (i) is small and positioned in the midline at the expense of the Shh-expressing floor plate (j). Shh expression in the notochord is unaffected in the mutant. Quantitation of data from 3 embryos/genotype and statistical analysis of data are presented in **S1 Table**.

We investigated the role of CCRK in Shh-dependent neural patterning through analysis of the *Ccrk* mutant spinal neural tube **(Figs 2 and S3)**. Shh signaling specifies a number of cell fates in the neural tube in a concentration and time-dependent manner [3]. The most ventral cell types, the floor plate and p3 interneuron progenitors flanking the floor plate, require the highest/most prolonged extent of Shh signaling. We found that the floor plate was absent in the *Ccrk* mutant neural tube, even though Shh expression in the notochord was maintained **(Figs 2J and S4)**. P3 progenitors, marked by Nkx2.2 expression, were reduced in number and located ectopically in the ventral midline of the mutants **(Fig 2I)**. The reduction in Nkx2.2+ cells was accompanied by ventral expansion of the Pax6-expressing domain in *Ccrk* mutants **(Figs 2F and S3H)**, as predicted by the established role of Nkx2.2 in repressing *Pax6* expression. Motor neuron progenitors (pMN) and their post-mitotic descendants, marked by Olig2, HB9, and Isl1/2 expression, require intermediate levels of Shh signaling while high levels of signaling inhibit their specification. In *Ccrk* mutants, the motor neuron progenitor domain expanded ventrally across the midline **(Figs 2G, 2H and S3F)**, suggesting that mutant cells exhibit intermediate responses to Shh even when they are positioned ventrally near the source of Shh ligand. Quantitation of the data (**S1 Table**) indicates that such changes are statistically significant. Analysis of patterning over developmental time (controlled for somite stage) revealed that the dorsalized phenotype was evident at the earliest stages of fate specification (10–13 somite stage), although by E11.5 (45–47 somite stage) the phenotype had partially recovered (**S4 Fig**). We also observed dorsal expansion of the Olig2, HB9, and Isl1/2 expression domains (motor neurons and their progenitors) in the *Ccrk* mutant neural tube (**Figs 2G, 2H and S3F**) in comparison to controls (**Figs 2B, 2C and S3E)**, which was statistically significant **(S1 Table**). The mutant phenotype suggests that *Ccrk* mutant neural progenitors in the midline fail to execute the strongest responses to Shh, whereas mutant cells in ventrolateral regions, dorsal to the normal motor neuron domain, exhibit higher than normal Hh pathway activity.

As *Shh* expression in the notochord appeared normal in *Ccrk* mutants, we hypothesized that CCRK is required for proper responses to the highest level of Shh signals, rather than production of Shh ligands. To address this, we performed a genetic experiment. Rab23 is an antagonist of the Shh pathway. In *Rab23* mutants, spinal neural progenitors adopt ventral cell fates, such as p3 and floor plate independently of Shh ligand or the pathway activator Smoothened [32, 34]. If CCRK acts in the pathway downstream of Shh ligand and of Smoothened, then the *Ccrk* mutant patterning phenotype would be genetically epistatic to that of *Rab23* mutants. Indeed, E10.5 *Rab23*^*-/-*^*Ccrk*^*-/-*^ double mutants exhibited loss of floor plate and reduction of p3 progenitor cell numbers. These features were indistinguishable from those in *Ccrk* single mutants, despite the loss of inhibition from Rab23 (**S5 Fig,** data quantitated in **S2 Table**). These data indicate that CCRK is important for regulating Hh pathway activity at a step downstream of Shh, Smoothened, and Rab23.

The slight dorsal expansion of the motor neuron domain in *Ccrk* mutant suggested that CCRK could also be required for complete repression of Hh pathway activity in the absence of signaling. *Smoothened* (*Smo*^-/-^) mutants are unable to respond to Hh ligands [35]. We reasoned that if loss of CCRK leads to mild, ligand-independent derepression of the Hh pathway, some Shh-dependent cell fates would be rescued in *Ccrk*^*-/-*^*Smo*^*-/-*^ double mutants. Indeed, we observed restoration of some Shh-dependent cell types, Olig2+ and Nkx6.1+ progenitors, in *Ccrk*^*-/-*^*Smo*^*-/-*^ double mutants, which were never seen in *Smo*^*-/-*^ single mutants (**S6O, S6P, S6K and S6I Fig;** data quantitated in **S3 Table)**. These data support an additional role for CCRK in complete repression of Hh pathway activity in the absence of Smoothened-dependent signaling.

### Genetic analysis of Gli transcription factor function in *Ccrk* mutants

Loss of *Gli2* results in a neural patterning phenotype similar to *Ccrk* mutants (loss of floor plate and reduction of p3 progenitors) [33]. If the *Ccrk* mutant neural patterning phenotype results primarily from loss of Gli2 function, then loss of Gli2 in *Ccrk* mutants should have little, if any, effect on their phenotype. However, analysis of *Ccrk*^*-/-*^*Gli2*^*-/-*^ double mutants revealed a much more dramatic dorsalization of neural tube progenitor identity than seen in either single mutant. (**S7K and S7I Fig**). Quantitation and statistical analysis of these data are presented in **S4 Table**.

Although Gli3 provides both positive and negative regulation of Hedgehog responses, its repressor function has a relatively dominant role in Hedgehog signaling [36]. In the spinal neural tube, the activator function of Gli3 is dispensable (due to redundancy with Gli2), whereas its repressor function is uniquely required. If *Ccrk* mutants retain at least some Gli2 function, then loss of the Gli3 repressor in this background should boost Hedgehog responses to provide phenotypic rescue. Indeed, *Ccrk*^*-/-*^*Gli3*^*-/-*^ double mutants showed phenotypic rescue in comparison with *Ccrk* mutants (**S8I and S8J Fig**, data quantified and analyzed in **S5 Table**). Given that *Gli2*^*-/-*^*Gli3*^*-/-*^ mutants show the opposite phenotype (loss of floor plate and p3 cell fates, [37]), we suggest that loss of CCRK partially impairs the functions of both Gli2 and Gli3 activators and that *Ccrk* mutants retain at least some Gli3 repressor function.

### CCRK regulates posttranslational modification of Gli proteins

**Fig 3:**
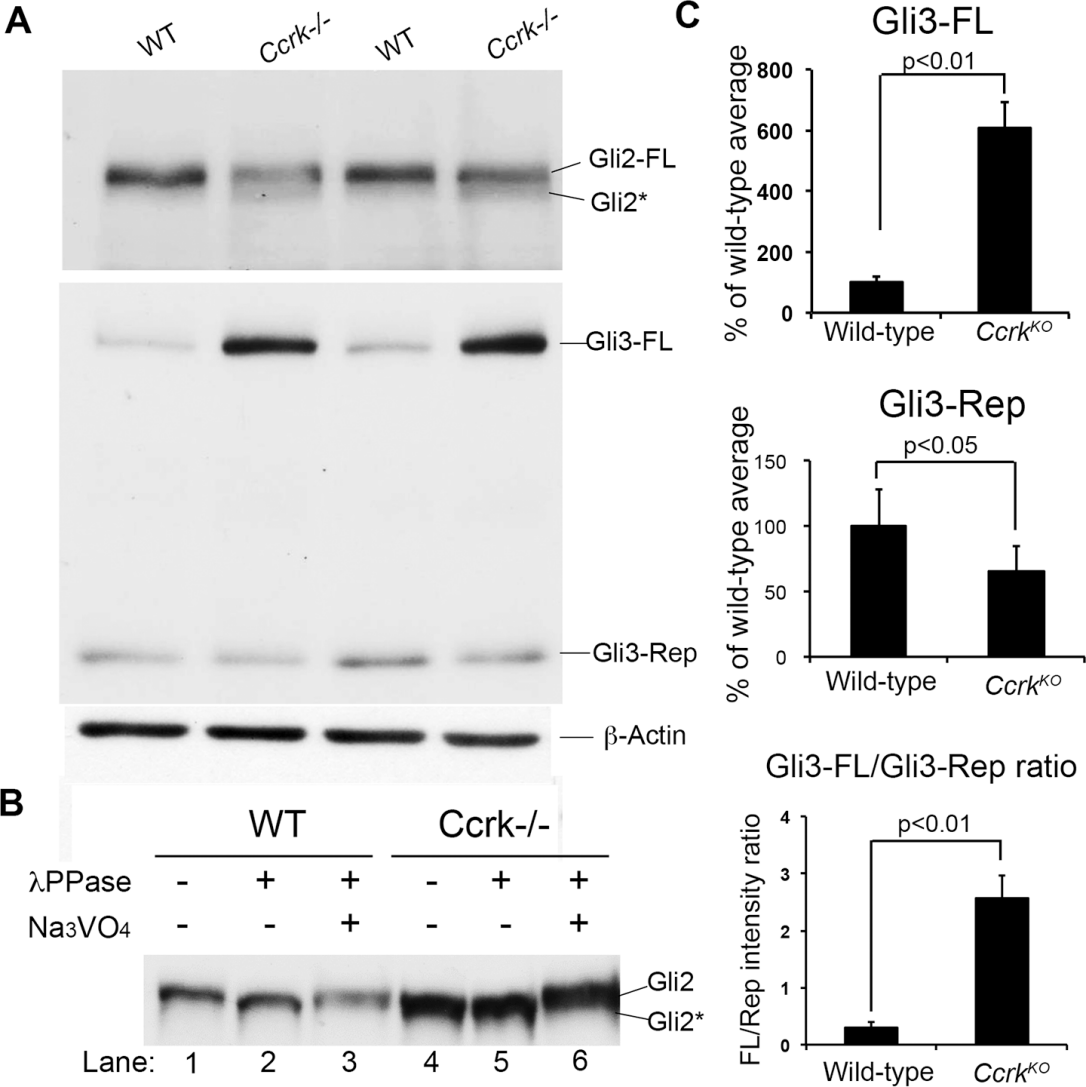
CCRK regulates Gli2 and Gli3 proteins. (A) Gli2 and Gli3 western blotting in E9.5 embryonic extracts; β-actin serves as a loading control. Full-length Gli2 (Gli2-FL) showed a second, faster migrating band (Gli2*) in *Ccrk* mutants, suggesting differential posttranslational modification. FL-Gli3 levels were significantly increased, whereas processed Gli3 repressor (Gli3-Rep) levels were somewhat reduced in *Ccrk* mutant extracts. (B) Extracts from wild-type and *Ccrk* mutant embryos were incubated alone, with addition of lambda phosphatase, or with lambda phosphatase plus the phosphatase inhibitor Na_3_OV_4_ and analyzed by Gli2 western blotting. The results suggest that the faster migrating species (Gli2*) from *Ccrk* mutants in (A) represents a dephosphorylated state. Similar results were observed in three separate experiments. (C) Quantitation of Gli3-FL, Gli3-Rep, and Gli3-FL/Gli3-Rep ratios, normalized to β-actin. 2 samples/genotype were analyzed. Error bars represent standard error of the mean (SEM). P values from Student’s t-tests are shown.

**Fig 4:**
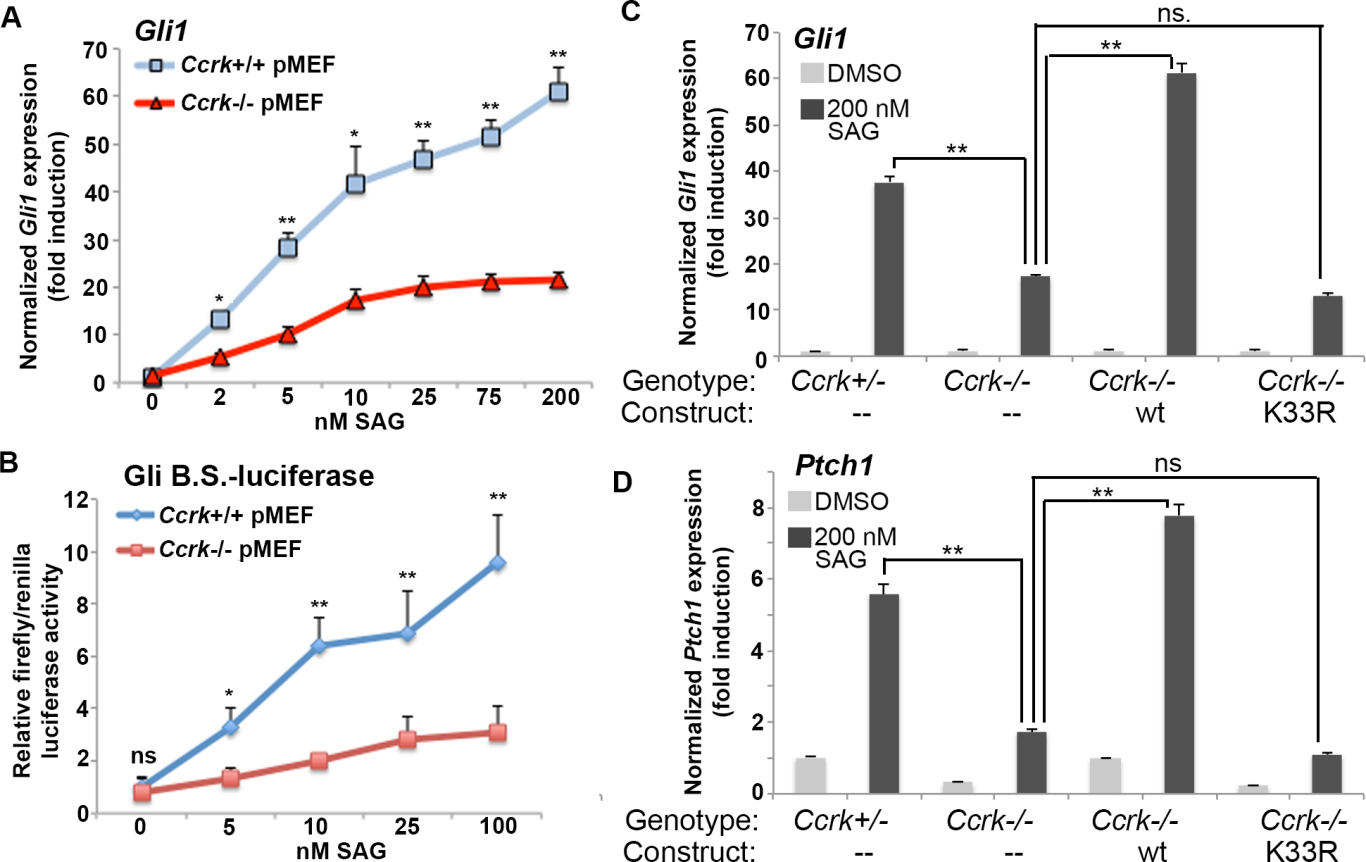
*Ccrk* mutant fibroblasts show diminished Hh pathway activity. (A) qPCR results for *Gli1* expression (normalized to *β-actin*) in wild-type and *Ccrk* mutant primary mouse embryonic fibroblasts (MEFs) stimulated with increasing concentrations of Smoothened agonist (SAG). (B) Normalized luciferase activity in immortalized wild-type and *Ccrk* mutant MEFs transfected with 8x Gli-binding site/firefly luciferase, as well as constitutive renilla luciferase, constructs and stimulated with SAG. (C, D) Normalized results of qPCR for *Gli1* (C) and *Ptch1* (D) expression in immortalized MEFs stably transfected with pcDNA3.1 (mock), wild-type CCRK, or K33R (kinase dead) mutant CCRK constructs in the presence or absence of 200 nM SAG. Error bars represent standard error of the mean. Data obtained from 3 biological replicates per condition. P values from Student’s t-tests: **, p<0.01; *, p<0.05; ns, not significant.

To complement our genetic analysis, we investigated how the regulation of Gli2 and Gli3 is affected at the protein level by disruption of *Ccrk*. We performed western blotting for Gli2 and Gli3 using extracts from E9.5 wild-type and *Ccrk* mutant embryos **(Fig 3A)**. Wild-type embryos showed a single full-length species of Gli2 (190 kDa), whereas in *Ccrk* mutant extracts we observed an additional, faster migrating species (**Fig 3A**). This pattern likely reflects a shift in posttranslational modification of Gli2, such as decreased phosphorylation, leading to diminished Gli2 activator function. We investigated this possibility and found that Gli2 mobility was increased in wild-type samples treated with lambda phosphatase, whereas Gli2 in *Ccrk* mutant samples showed increased mobility similar to phosphatase treated wild-type samples (lanes 2 and 4 in **Fig 3B**) even without phosphatase treatment. This suggests that Gli2 in mutant cells occurs in a hypo-or non-phosphorylated state. As expected, inclusion of the phosphatase inhibitor Na_3_VO_4_ reversed the effect of phosphatase on Gli2 in wild-type samples (**Fig 3B**, lane 3). Curiously, Na_3_VO_4_ treatment caused Gli2 migration to be retarded in *Ccrk* mutant samples (Fig 4B, lane 6). These results were consistently observed in three separate experiments. Our interpretation of the results is that, during incubation of extracts Gli2 is phosphorylated and dephosphorylated by endogenous kinases and phosphatases (when lambda phosphatase and Na_3_VO_4_ were omitted). It appears that the balance of these activities favors phosphorylation in wild-type samples and dephosphorylation in *Ccrk* mutant samples.

We also observed profound effects of the *Ccrk* mutation on the abundance of Gli3 forms. In wild-type embryos, Gli3 is found in two forms: a full-length form (185 kDa) that can be rendered into the activator state (Gli3Act) and a proteolytically generated 85 kDa repressor form (Gli3Rep) [5]. Shh signaling suppresses formation of Gli3Rep in favor of the full-length form that can function as a transcriptional activator. We found that the full-length form of Gli3 was significantly increased (~4-fold) in *Ccrk* mutant embryos (**Fig 3C**). Despite this increase, our genetic data indicate that the Gli3 activator function is impaired in *Ccrk* mutants given that *Ccrk/Gli2 and Gli2/Gli3* double mutants show a similar, strongly dorsalized, neural patterning phenotype (**S7 Fig**, [37]). The increase in full-length Gli3 levels in the mutants may reflect increased stability of non-functional full-length Gli3 (see Discussion). We also observed a modest (~40%) decrease in the levels of the Gli3 repressor form in *Ccrk* mutants, which may reflect inefficient proteolytic processing of Gli3 and may explain the weak ligand-independent activity of the Hedgehog pathway in *Ccrk* mutants indicated by the partial rescue phenotype of *Smo/Ccrk* mutants (**S6 Fig**). Collectively, the data indicate that CCRK is required for proper posttranslational modifications of Gli2 and Gli3, necessary for their functions as transcriptional regulators.

### *Ccrk* mutant cells show impaired Hedgehog pathway responses *in vitro*

To investigate the defect in Hedgehog signaling in *Ccrk* mutants in more detail, we generated primary mouse embryonic fibroblasts (pMEFs) from *Ccrk* mutant and control embryos. We compared Hh pathway activity between mutant and wild-type cells in unstimulated cells, as well as in cells stimulated with Smoothened agonist (SAG). When exposed to varying concentration of SAG for 24 hours, the direct Hh target gene *Gli1* (monitored by qPCR) was expressed at a reduced level (30–40%) compared to wild-type cells (**Fig 4A**). This phenotype was confirmed using wild-type and mutant cells transfected with a Gli binding site/luciferase reporter (**Fig 4B**).

We next investigated immortalized wild-type and *Ccrk* mutant MEF cell lines. We validated this model by stably transfecting the mutant cell lines with constructs overexpressing either wild-type CCRK or a version with a K33R mutation that is predicted to disrupt kinase activity [29]. The immortalized *Ccrk* mutant cells showed a deficiency in Hh pathway activity similar to primary *Ccrk* mutant cells (**Fig 4C and 4D**). Mutant cells harboring the wild-type construct showed responses that were slightly higher than controls, whereas mutant cells harboring the K33R mutant version showed no evidence of rescue. These data confirmed the role of CCRK in the phenotype and indicated that the kinase activity of CCRK is required for its ability to potentiate Hh pathway activity.

**Fig 5:**
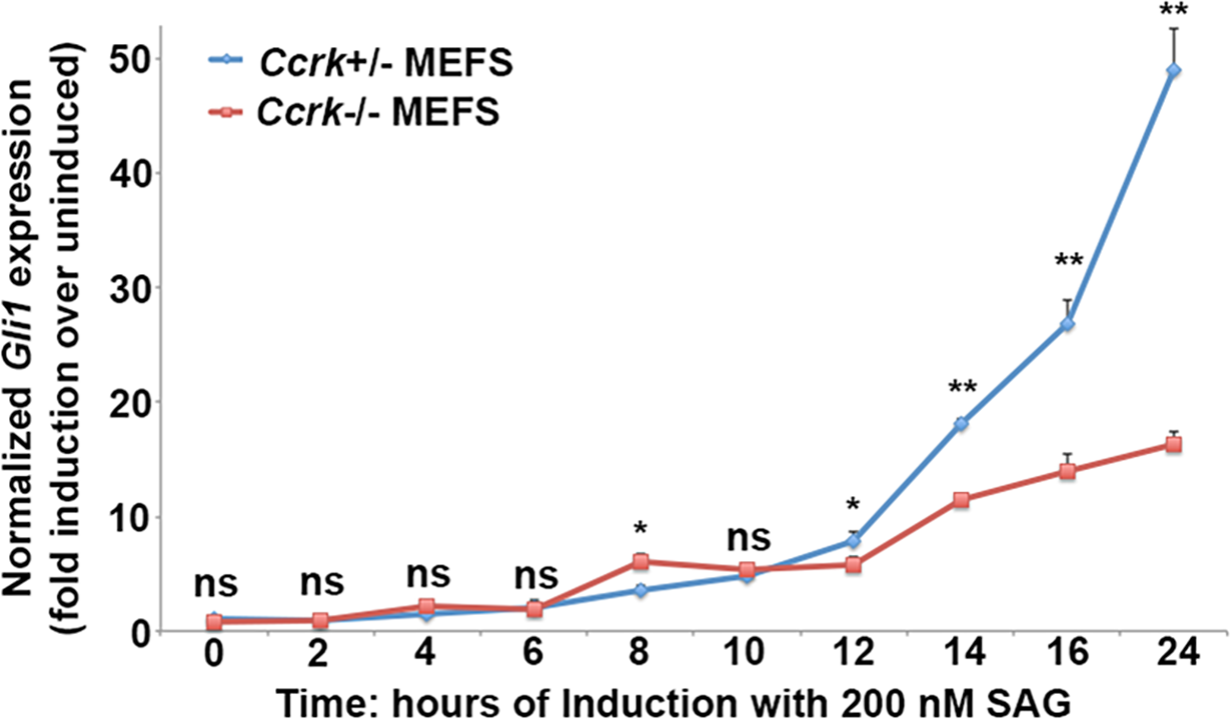
*Ccrk* mutant cells show defective long-term Hh pathway responses. *Ccrk* mutant and control MEFs treated with 200nM SAG for varying periods of time (0 to 24 hours). Normalized *Gli1* expression (fold induction over unstimulated cells) assayed by qPCR is shown. Note that the deficit in mutant responses is seen at late time points (starting at 12 hours treatment). Error bars reflect standard error of the mean. Data obtained from 3 biological replicates per condition. P values from Student’s t-tests: **, p<0.01; *, p<0.05; ns, not significant.

Next, we investigated the defect in Hh pathway activity as a function of time of SAG exposure. We stimulated cells with 200 nM SAG for periods ranging from 2 to 24 hours. Interestingly, we found normal or slightly elevated levels of induction of *Gli1* expression in *Ccrk* mutant cells up to 12 hours of SAG exposure in comparison to control cells (**Fig 5**). However, after about 12 hours of exposure, wild-type cells exhibited continuously increased levels of expression, whereas the increase was modest for *Ccrk* mutant cells. A similar phenomenon was observed when cells were stimulated with lower amounts of SAG (5 or 20 nM, **S9A Fig**). *Ccrk* mutant cells showed a qualitatively similar pattern of responses with respect to *Ptch1* expression, although expression by mutant cells was slightly reduced at times ≤12 hours with larger differences in expression seen at 14–24 hours (**S9B Fig**). These results suggest that CCRK function is more important for later periods of signaling than for short-term responses.

Because wild-type cells show a continuous increase in *Gli1* expression with increased duration of SAG exposure, it is possible that the activity at 24 hours represents prior pathway activity combined with recent pathway activity. If this were the case, the defective responses in *Ccrk* mutant cells at 24 hours exposure could be due to a loss in their memory of prior activity or to a failure to mount strong responses beyond 8 hours of exposure. To investigate this, we conducted a hysteresis experiment to determine to what extent the cells retain activity from prior signaling events once the inducer is removed for extended periods (**S10 Fig**). When wild-type and mutant cells were exposed to SAG for 8 hours, they mounted similar responses. When cells were treated with SAG for 8 hours, followed by SAG washout (plus inclusion of the Smo inhibitor Cyclopamine) for 24, 48, or 72 more hours, wild-type cells showed levels of *Gli1* expression similar to, or somewhat higher than, those observed immediately after 8 hours of SAG exposure, indicating that prior exposure to SAG maintains *Gli1* expression for an extended period. The *Ccrk* mutant cells also retained wild-type levels of *Gli1* expression 24 hours after removal of inducer, although their responses were somewhat lower than wild-type at 48 and 72 hours. Collectively, these data argue that the failure of *Ccrk* mutant cells to mount strong responses to exposure times beyond 8 hours lies in their failure of long-term signaling rather than a loss of memory of prior signaling events.

### CCRK regulates the morphology and length of primary cilia

**Fig 6:**
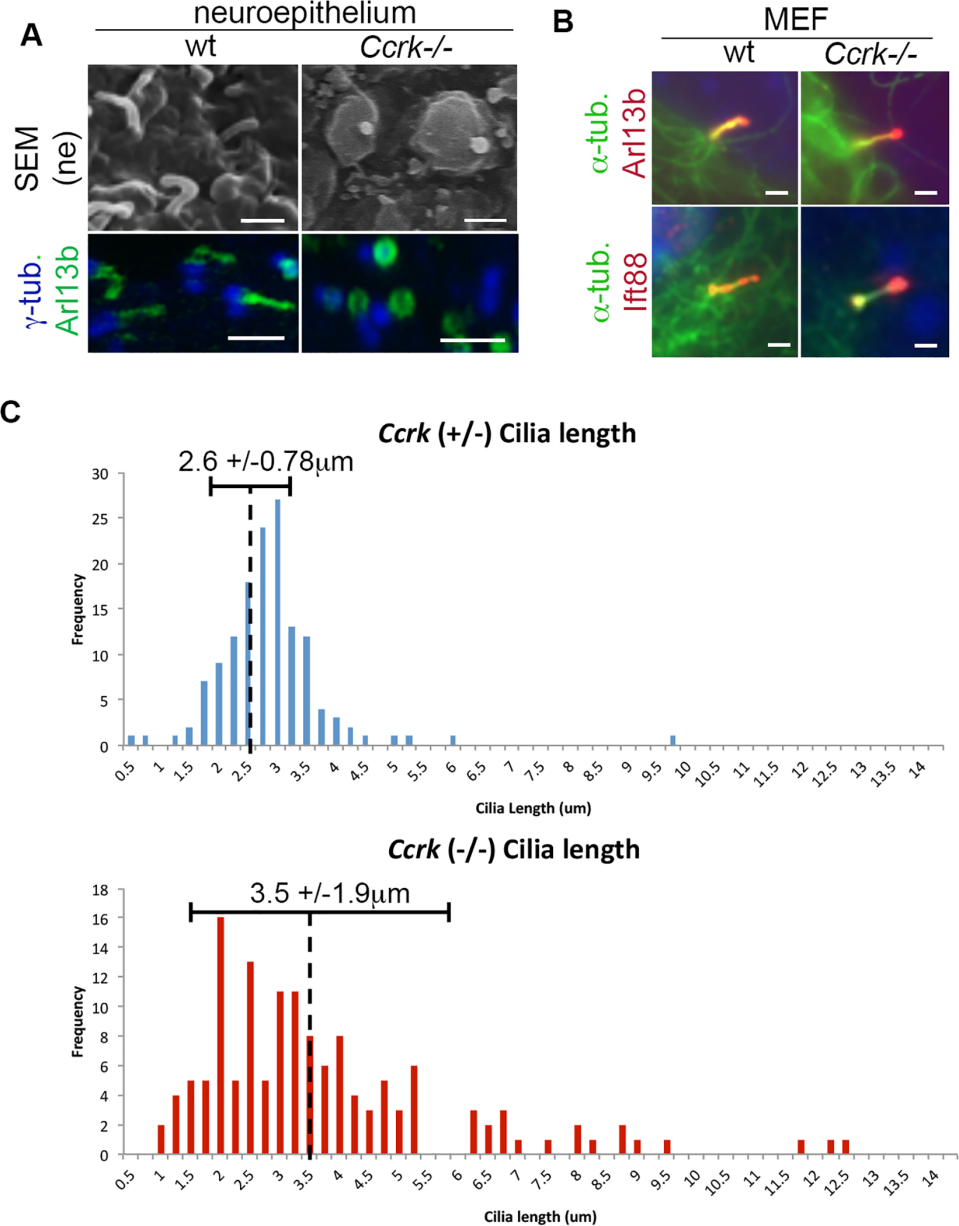
Regulation of ciliary morphology and length by CCRK. (A) Primary cilia in the E9.5 *Ccrk* mutant and wild-type neuroepithelium assayed by scanning electron microscopy and Arl13b/gamma-tubulin staining. (B) Primary cilia in *Ccrk* mutant and wild-type MEFs stained with antibodies against acetylated α-tubulin, Arl13b, and IFT88. Scale bars in A and B are 1 μm. (C) Distribution of ciliary length in *Ccrk* mutant and control (*Ccrk*^*+/-*^) MEFs binned in 0.25 μm groups. Lengths were measured from Arl13b and γ-tubulin-stained cilia. Average lengths and standard deviation for distributions are shown.

Because CCRK homologs in other organisms function in ciliogenesis and because ciliary defects were observed in mutants for the CCRK-binding protein BROMI, we investigated the effects of CCRK disruption on cilia in the mouse. Cilia in the *Ccrk* mutant embryonic neuroepithelium were generated at roughly normal frequencies but they adopted a shortened, swollen morphology (**Figs 6A and S11**). Staining with the ciliary marker Arl13b showed a small ring-like pattern (**Fig 6A**). Both of these ciliary phenotypes resemble those found in *bromi* mouse mutants [23]. We next analyzed cilia in *Ccrk* mutant and control MEFs. *Ccrk* mutant MEFs generated cilia, albeit with notable differences from wild-type. First, we found that, while both Arl13b and the IFT protein IFT88 localized to *Ccrk* mutant cilia, they showed an accumulation at the distal end of the cilia in comparison to wild-type cilia (**Fig 6B**). This phenotype suggests an imbalance between the processes of anterograde and retrograde IFT and is similar to the distal swelling seen in *Chlamydomonas* flagella null for the *Ccrk* homolog *lf2* [29]. Second, we measured the length of wild-type and *Ccrk* mutant cilia under steady-state (serum starved) conditions (**Fig 6C**). Although the average cilia lengths were generally similar (2.6 and 3.5 μm, for control and mutant cilia, respectively), there was a broader distribution of cilia lengths in the mutant cells than in the control cells (std. dev. of 0.78 vs. 1.9 for control and mutant cilia, respectively). This finding is reminiscent of the length phenotype of *Chlamydomonas lf2* null flagella, which can be much longer or shorter than those of wild-type [29].

**Fig 7:**
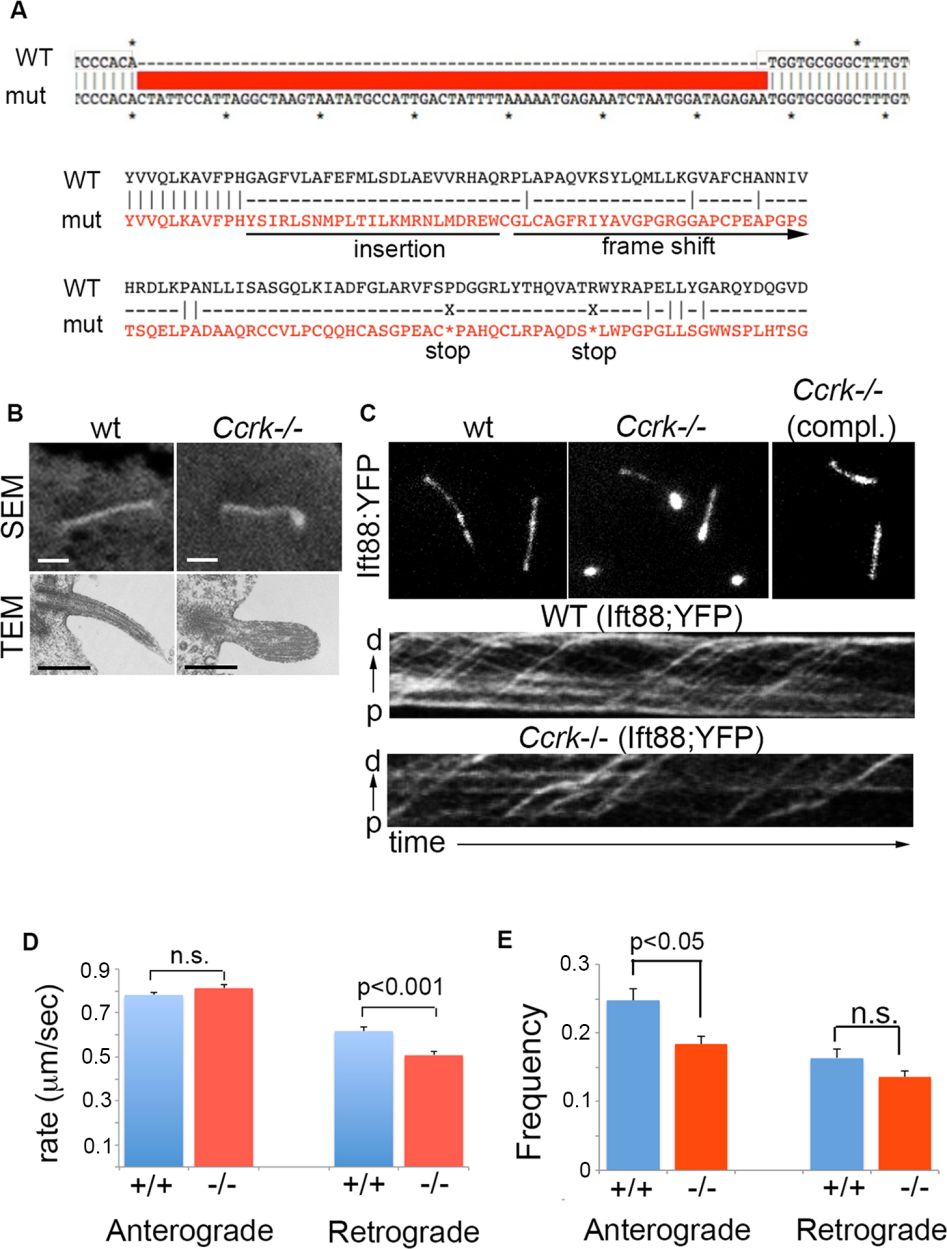
Ciliary phenotypes and IFT assays in wild-type and *Ccrk* mutant IMCD3 cells harboring an IFT reporter. (A) *Ccrk* mutant IMCD3 cells (expressing an IFT88::YFP construct)were generated by CRISPR/Cas9 mutagenesis. A clone with a homozygous 67 bp insertion (causing a frame shift and multiple nonsense codons downstream) was identified and analyzed further. (B) Scanning and transmission electron microscopic analysis of wild-type and *Ccrk* mutant IMCD3 cilia. The mutant cilia showed swelling at their distal ends. (C) IFT88::YFP expression in wild-type and *Ccrk* mutant IMCD cells. The *Ccrk* mutant cells uniformly showed accumulation of IFT88::YFP at their distal ends. Transfection of the mutant cells with a wild-type *Ccrk* expression construct rescued this phenotype. Kymographs from live imaging of IFT88::YFP particle movement in wild-type and *Ccrk* mutant cilia are shown (distal cilia ends at the top, time increases from left to right). Anterograde transport occasionally showed abrupt changes in speed in the mutants. Rates (D) and frequencies (E) of anterograde and retrograde IFT particle movement were determined from the kymographs. Speed quantitation was determined from 235 and 61 particles (anterograde) and 74 and 25 particles (retrograde) in wild-type and *Ccrk* mutant cilia, respectively. Frequency quantitation was determined from 45 and 64 cilia movies from wild-type and *Ccrk* mutant cells, respectively. Error bars represent standard error of the mean. Statistical analysis represents results from Student’s t-test. Scale bars in B are 1 μm.

We also interrogated the role of CCRK in a cell type that generates longer cilia, IMCD3, engineered to express a fluorescent reporter, IFT88::YFP [38], allowing us to monitor intraflagellar transport. We used CRISPR/Cas9-mediated mutagenesis to disrupt *Ccrk* in IMCD IFT88::YFP cells (**Fig 7A**). *Ccrk* mutant IMCD cells generated cilia but, similar to neuroepithelial cells and MEFs, the mutant cilia showed swelling at the distal ends, as determined by scanning and transmission electron microscopy (**Fig 7B**). This phenotype was accompanied by the accumulation of IFT88::YFP at the distal ends of cilia (**Fig 7C**). Stable transfection of the *Ccrk* mutant IMCD cells with a construct expressing wild-type CCRK resulted in complementation/phenotypic rescue. We next monitored the movement of IFT88::YFP particles in live wild-type and *Ccrk* mutant IMCD cells using TIRF microscopy to generate kymographs from which we determined rates of anterograde and retrograde IFT (**Fig 7C**). The rates of anterograde transport were not statistically different between wild-type and mutant cells. Retrograde IFT occurred at a slightly slower rate in *Ccrk* mutant cells compared to controls (0.62 and 0.51 μm/sec, in wild-type and mutant cells, respectively, **Fig 7D**). Although it was difficult to determine IFT frequencies due to movie-to-movie variability, IFT events occurred at normal or somewhat reduced frequencies in the mutant cells (**Fig 7E**). These data indicate that IFT was only modestly affected in *Ccrk* mutant cells, a result similar to findings from *Chlamydomonas lf2* mutants [39]. While this defect may have some contribution to the *Ccrk* mutant ciliary length and morphology phenotype, other deficits, such as transport of the ciliary cargo *per se*, may play a more important role.

### CCRK controls the kinetics of ciliary protein localization

**Fig 8:**
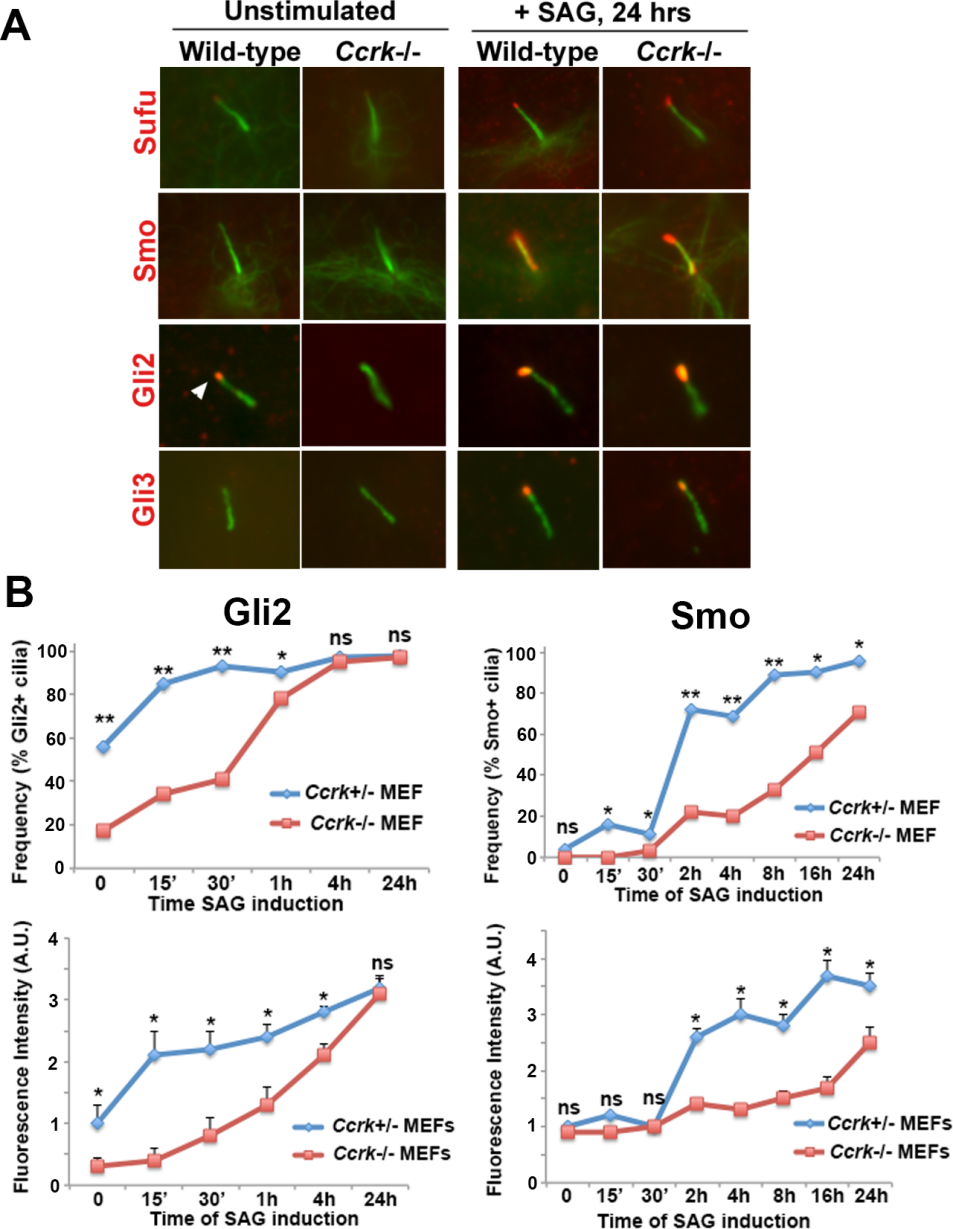
Localization of Hedgehog signaling proteins to *Ccrk* mutant cilia. (A) Localization of Hh pathway components to wild-type and *Ccrk* mutant MEFS under unstimulated or stimulated (200nM SAG for 24 hours) conditions. Note that Gli2 staining (arrowhead) was rarely observed in *Ccrk* mutant cilia under unstimulated conditions. (B) Ciliary localization of Gli2 and Smo was monitored in terms of frequency and staining intensity as a function of time of SAG exposure (0 to 24 hours). Percentages of cilia with Gli2 or Smo localization were determined from between 100 and 300 cilia per condition and statistical significance was analyzed using Chi-square tests. Staining intensity was measured from between 25 and 40 cilia per condition and statistical significance was analyzed using Student’s t-tests. Error bars represent standard error of the mean. P values from statistical analyses: **. P<0.01; *, p<0.05; ns, not significant.

We determined whether the localization of Hedgehog signaling components to *Ccrk* mutant cilia was affected in embryonic fibroblasts. We assayed the localization of SuFu, Smo, Gli2 and Gli3 under unstimulated conditions or after 24 hours of stimulation with the Smoothened agonist. Each of these proteins shows enrichment in cilia upon Hh pathway stimulation [17, 18]. We observed similar patterns of localization for these proteins under the two steady-state conditions in wild-type and *Ccrk* mutant cells (**Fig 8A**). However, Gli2 localized to wild-type cilia more frequently than to mutant cilia under unstimulated conditions. We investigated the temporal change in localization patterns by assaying the frequency and amount of ciliary Gli2 localization as a function of time of SAG-mediated pathway induction. Interestingly, the frequency of wild-type cilia with Gli2 localization reached a plateau around 30 min of induction, whereas *Ccrk* mutant cilia did not show uniform localization until 4 hours of induction (**Fig 8B**). Similarly, the kinetics of increase in ciliary Gli2 fluorescence upon induction were much slower in *Ccrk* mutant cells than in wild-type cells. Analysis of Smoothened ciliary localization revealed a similar delay in recruitment in *Ccrk* mutant cells. These data suggest that, although the rates by which IFT complexes move are relatively unaffected by loss of CCRK, the efficiency of ciliary recruitment and transport of Hedgehog regulatory proteins, and perhaps other cargo, is controlled by CCRK. These findings suggest that CCRK regulates ciliary length, morphology, and function in Hedgehog signaling by promoting the efficiency of ciliary cargo recruitment and transport.

## Discussion

We show here that the evolutionary conserved kinase, CCRK, regulates a broad array of processes at levels ranging from the subcellular to the organismal. We find that mouse CCRK controls several aspects of ciliogenesis, which in turn are important for Hedgehog signal transduction, patterning, and embryonic morphogenesis. The role of *Ccrk* is similar to that of *broadminded* (*bromi)*, identified in a forward genetic screen [23]. BROMI protein physically associates with CCRK and promotes its stability. In support of a common function, *Ccrk* null mutants phenocopy *bromi* mutants with respect to embryogenesis, Hedgehog pathway effects, and ciliary defects.

We suggest that CCRK in the mouse plays a role analogous to LF2, its homolog in the green algae *Chlamydomonas reinhardtii*. *Chlamydomonas* flagella show a structure strikingly similar to that of mammalian cilia and both organelles are assembled using similar mechanisms, such as intraflagellar transport (IFT). A comparison of the phenotypes of *Chlamydomonas lf2* null mutants with those of *Ccrk* null mutant cells shows close similarities. First, *Ccrk* and *lf2* null cells both generate cilia/flagella with a broader range in lengths compared with controls. Second, similar to *lf2* mutant flagella, mouse *Ccrk* null cilia show swelling at their distal ends, which is accompanied by an accumulation of IFT proteins at this site. Third, recent analysis of *lf2* mutant flagella undergoing regeneration revealed that IFT particles loaded with ciliary cargo (tubulin) were transported much less frequently than controls, although the transport frequency of all IFT particles (loaded or unloaded) was only slightly reduced [39], suggesting that these mutants fail to load IFT particles with cargo properly or to dispatch loaded particles from the flagellum base. Similarly, *Ccrk* mutant cilia show only small changes in the rates and frequencies of IFT, whereas the enrichment of cargo (Smoothened, Gli2) in cilia upon Hh pathway activation was substantially slower in *Ccrk* mutant cells.

### CCRK regulation of ciliary length and structure

*Ccrk* mutant cilia show two overt phenotypes: swelling at their distal ends and defective length control. The distal swelling is accompanied by accumulation of the ciliary proteins Arl13b and IFT88 at this end. Such a phenotype is consistent with a defect in retrograde IFT or, possibly, an imbalance in the efficacy of retrograde versus anterograde IFT [14, 15]. The slightly reduced retrograde IFT rates and frequencies in *Ccrk* mutant cilia may be responsible for this phenotype. Alternatively, a defect in the IFT turnover process at distal ends of cilia, which involves disassembly/unloading of anterograde IFT trains and assembly/loading of retrograde IFT trains, could lead to such a phenotype. CCRK could regulate the activity of retrograde IFT machinery *per se* or it could control the ciliary import of proteins that are rate-limiting for retrograde transport.

The role of CCRK in the control of ciliary length is less clear, as the means by which cells sense and control their lengths are not well understood. Cilia and flagella initially undergo a lengthening phase, then slow their growth to reach a steady-state length. Many models have been proposed to explain how cells sense their ciliary length, such as the “time-of-flight” or ion current models [40]. Regardless of the sensing mechanism, the cell must execute some form of length control in response to this information. We suggest that differential loading of cargo onto IFT particles provides this control [39, 41]. According to this hypothesis, during a growth phase, IFT particles are efficiently loaded with cargo needed to assemble cilia at their tips. However, as the cilium begins to reach a steady-state length, cargo loading is significantly down-regulated, leading to reduction in the amount of ciliary cargo being delivered. If cargo, such as tubulin, were rate-limiting for ciliary assembly, this would cause slowing and then stopping of ciliary growth. Thus, ciliary cargo loading may be improperly regulated in *Ccrk* mutant cells and the variation in ciliary length may be explained by such a defect. In *Chlamydomonas* IFT trains loaded with tubulin are transported frequently during the growing phase, but this frequency is strongly attenuated when the cilium is at steady state. In *lf2* mutants with regenerating flagella, the frequency of transport is greatly reduced compared to controls. In these mutant flagella, at steady state, the frequency is higher than in normal flagella. Reduced transport during growth delivers fewer tubulin subunits to the cilium, resulting in its shortening in some cells. However, in other *lf2/Ccrk* mutant cells, there may be a delay in reaching steady state, as cargo loading and transport is not appropriately down-regulated, causing those cilia to grow longer than normal.

### Regulation of Hedgehog signaling by CCRK in the embryo

Disruption of *Ccrk* in the embryo results in phenotypes indicative of defects in Hedgehog signaling, similar to other mouse mutants with elevated or decreased activity of the pathway [31–33]. In *Ccrk* mutants, cell fates in the most ventral neural tube, that require strong Shh signaling, are either absent (floor plate) or reduced in number and mispositioned (p3 cells), while cell fates requiring intermediate levels (pMN cells) are concomitantly expanded into ventral territories. This suggests that, while *Ccrk* mutants are capable of Shh signaling, the strongest responses are defective, with cells executing intermediate responses instead. In addition, the *Ccrk* mutant pMN domain also expands dorsally, suggesting that cells that would normally adopt fates controlled by low levels of signaling behave as if they experience intermediate levels. The observation that cells requiring weak activity of the pathway are restored in *Smo* mutants when *Ccrk* is mutated suggests that CCRK represses the Hh pathway in the absence of signals. Thus, CCRK both potentiates and represses the activity of the Hh pathway, depending on context.

The *Ccrk/Rab23* epistasis data, along with the SAG *in vitro* data, indicate that CCRK acts in the response to Shh (rather than in ligand production) at the level of Smo or downstream of it. Hh signaling in the mouse embryo is mediated by the combined functions of Gli2 and Gli3 activator/repressor forms. The epistasis and biochemical analyses suggest that all of these activities are partially impaired in *Ccrk* mutants. Despite the similarity between the *Ccrk* and *Gli2* mutant patterning phenotypes, the *Ccrk* mutant phenotype is not caused by loss of Gli2 activity alone, as disruption of *Gli2* in *Ccrk* mutants resulted in exacerbation of the phenotype (stronger dorsalization) and because disruption of *Gli3* in *Ccrk* mutants resulted in phenotypic rescue, whereas disruption of Gli2 and Gli3 results in a strongly dorsalized patterning phenotype [37]. It is unlikely that the *Ccrk* mutant phenotype is due to loss of Gli3Act function alone, as Gli3 activator is not required for floor plate specification in the presence of Gli2. Thus, the most parsimonious explanation for these data is that both Gli2Act and Gli3Act functions are impaired in *Ccrk* mutants and this has the combined effect of diminishing strong Shh responses. Low-level Smo-independent Hh pathway activity in *Ccrk* mutants might be due to reduced Gli3Rep function. Indeed, the mutants exhibited reduced amounts of the 85 kDa Gli3Rep form, consistent with diminished proteolytic processing of the Gli3. However, the residual Gli3Rep in these mutants is likely to be functional since loss of *Gli3* can rescue the *Ccrk* mutant phenotype.

The state of Gli2 and Gli3 proteins in *Ccrk* mutant embryos supports the arguments presented above. The full-length (FL) forms of both Gli2 and Gli3, representing GliAct or GliAct precursor forms, are affected in the mutants. FL-Gli2 changes its electrophoretic migration, consistent with inefficient post-translational modification (e.g., phosphorylation). The change could represent incomplete PKA phosphorylation at the Pc-g sites in the N-terminus of Gli2, which potentiate Gli2Act by promoting its dissociation from Sufu [7]. FL-Gli3 shows dramatic accumulation in *Ccrk* mutants. Although this could, in principle, cause hyperactivation of Shh responses in *Ccrk* mutants, it may also be a secondary effect of reduced Gli3Act function. This is because release of full-length Gli proteins from their inhibitor, Suppressor of Fused (Sufu) [6], leads to rapid degradation of such proteins, whereas failure to dissociate from Sufu leads to FL-Gli stabilization but loss of its transcriptional functions [42].

Collectively, the genetic and biochemical data suggest that the functions of Gli2Act and Gli3Act forms are impaired, possibly due to their inability to efficiently dissociate from Sufu. In addition, inefficient processing of FL-Gli3 results in diminished Gli3Rep function, which, in turn, increases inappropriate, ligand-independent activation of the Shh pathway.

### Loss of CCRK causes ciliary and Hh signaling defects that both resemble and differ from those resulting from loss of other ciliary regulators

While the ciliary and Hh signaling phenotypes of *Ccrk* mutants share some similarities with those of other mouse mutants with mutations in ciliary regulators, they differ in important ways from each. Loss of IFT B proteins (such as Ift172), like loss of *Ccrk*, results in both diminished high level Hh responses and weak ectopic Hh pathway activity. However, IFT B mutants typically fail to generate cilia (unlike *Ccrk* mutants) and the intermediate/high level Hh responses are more significantly impaired in IFT B mutants than *Ccrk* mutants [13]. Loss of IFT A proteins (such as Ift122) or cytoplasmic dynein (Dync2h1) result in accumulation of IFT complexes in the distal tips of cilia, similar to *Ccrk* mutants. However, loss of IFT A proteins only causes increased Hh pathway activity, *Dync2h1* mutants only show diminished pathway activity, whereas *Ccrk* mutants show both diminished and ectopic pathway activity [14, 15]. Although loss of the ciliary kinesin Kif7 and loss of CCRK each cause deregulation of ciliary length, their effects on Hh pathway activity and neural patterning are largely opposite from one another [43, 44]. Thus, it appears that the roles of CCRK in ciliary assembly and Hh pathway control are distinct from those of previously studied regulators, although CCRK and such regulators are likely to be functionally connected.

### Regulation of Hedgehog signaling components by CCRK in the cilium

We suggest that CCRK indirectly controls the function and biochemical state of Hh signaling components by promoting their import into the cilium. This is supported by our analyses of Smo and Gli2 ciliary localization. In normal cells during the first several hours of SAG exposure, the levels of Smo and Gli2 increase in the cilium over time indicating the rate of ciliary import of these proteins exceeds the rate of their ciliary export during the first 1–2 hours of signaling. In contrast, the rates of Gli2 and Smo ciliary accumulation were substantially delayed in *Ccrk* mutant cells. This observation may be explained by a reduced rate of ciliary import of Gli2 and Smo. Alternatively, there may be an increase in the rate of ciliary export of these factors in *Ccrk* mutant cells. While we cannot definitively distinguish between these possibilities at present, we favor the first one. The distal accumulation of Gli proteins in retrograde IFT mutant cilia [15], coupled with direct interactions between Gli proteins and Kinesin II subunits [45], suggest Gli proteins are transported by IFT. Because retrograde IFT rates are modestly impaired in *Ccrk* mutant cilia and the frequency of IFT events is somewhat reduced in such cilia, an increase in Gli2 ciliary export is unlikely. The accumulation of IFT88 at the distal ends of *Ccrk* cilia is also consistent with impaired retrograde IFT. Although we were only able to assay the change in cargo accumulation during the first few hours after stimulation (as the levels of proteins reach a steady state thereafter), we hypothesize that *Ccrk* mutant cilia continuously exhibit reduced flux of cargo transport. A reduction in the rate of ciliary transport of Hh pathway components could result in diminished Hh pathway activity. Signaling downstream of Smo results in modification of Gli proteins and dissociation from their inhibitor Sufu, events which are dependent on their transport within cilia [20, 21]. Thus, diminished influx of pathway activators (Smoothened and Gli2) could result in the generation of fewer activated Gli transcription factor molecules per unit of time. This would cause a cumulative defect in the transcriptional output of the pathway.

### Regulation of Hh pathway responses over time

Examination of *Gli1* induction in wild-type cells over time suggests there are at least two phases in the response. During the first phase, between 2 and 12 hours, the increase in target mRNA level is mostly linear with a shallow slope with respect to induction time. This could be explained by the generation of a fixed level production of target gene message per unit of time coupled with perdurance of previously made transcripts. During the second phase, between 12 and 24 hours of exposure, the magnitude of the response increases much more rapidly. The change of slopes is related to the state of the network during each of these periods.

The change in the rate of target mRNA increase between the first and second phases may be explained by recruitment of a signal amplification mechanism to further enhance responses during the second phase. While we do not know the mechanistic basis of this amplification, it may rely on the production of more *Gli2* which is transcriptionally up-regulated by Shh signaling in fibroblasts [46]. Thus, the time of onset of this amplification would be determined by the time it takes from the initial increase in *Gli2* transcription to the generation of active Gli2 protein. Once active Gli2 protein levels accumulate, they may mediate a more robust response to SAG per unit of time.

In *Ccrk* mutant cells, the rate of increase in *Gli1* and *Ptch1* expression with SAG exposure remains slow and continuous from 2 hours until well beyond 24 hours of induction, suggesting that the sensitization mechanism normally acting during the second phase is defective. The hysteresis experiments indicate that the dampened responses of mutant cells at late time points are caused by a reduction in the generation of activated Gli proteins per unit of time, rather than loss of cellular memory of prior signaling events (due to, e.g., shortened half-lives of activated Gli proteins).

We suggest a model (**S12 Fig**) in which CCRK increases the rate of import of Hh pathway components during pathway stimulation. However, the decreased rate of cargo import in *Ccrk* mutant cells does not impact the ability of such cells to mount appropriate responses during the first phase of signaling. In contrast, the reduced import rate is limiting with respect to mounting an amplified response during the second phase. If the amplification mechanism occurs through increasing Gli2 protein levels, it is possible that the additional Gli2 critically depends on its efficient transport into and out of the cilium.

While our model is testable, it remains unclear how the import of ciliary cargo is regulated and what the role of CCRK in this mechanism may be. Future identification of CCRK substrates should be helpful in this regard.

## Materials and methods

### Ethics statement

All experiments on mice were conducted according to institutional and NIH animal welfare regulations. Mice were euthanized according to AVMA approved guidelines (CO_2_ inhalation). Animal experiments were approved by the IACUC committee at the University of Georgia (Approval #A2012 12-009-A2).

### Generation of a Ccrk null mutant allele

A bacterial artificial chromosome clone containing the *Ccrk* gene generated from mouse AB2.2 embryonic stem (ES) cell genomic DNA was used for targeting construct design. The clone was modified by two homologous recombination steps in *E*. *coli* using a recombineering method (Red/ET kit, GeneBridges). A *LoxP* site was inserted 1020 bp upstream of the first exon of *Ccrk*, and a *LoxP-PGK*::*neo*^*R*^*-LoxP* cassette was inserted 430 bp downstream of the end of exon 2 (within intron 2) of *Ccrk*. A 12.5 kb Xba1 fragment (harboring a 6.85 kb 5′ arm of homology and a 2.55 3′ arm of homology) was subcloned into pBluescript. The XbaI fragment was then isolated from the vector backbone and used for homologous recombination in ES cells.

AB2.2 mouse ES cells were grown on feeder cells, electroporated with the targeting construct, and selected with G418 following standard protocols [47]. Targeted clones were identified by PCR using a forward primer within the *neo*^*R*^ sequence and a reverse primer positioned within the genomic sequence 245 bp downstream of the end of the 3′ arm of homology. Targeting was verified by PCR using primers at the 5′ and 3’ ends of homology and euploidy confirmed [47]. Injections of targeted ES cells resulted in several chimeric animals that transmitted the allele, *Ccrk*^*fl*^, through the germline. *Ccrk*^*fl*^/+ males were crossed with *Tg(EIIa-Cre)*/+ deleter females (Jackson labs) to generate the null allele of *Ccrk*. The progeny was screened for Cre-mediated excision between the 5’-most and 3’-most *LoxP* resulting in the *Ccrk*^*KO*^ mutant allele, harboring a deletion of exons 1 and 2 and carrying a single LoxP site using primer pairs for the wild-type (GCAGGAGCCTATGCTGGATCCCTGT and AACGATCTCGCCAGTCTGTGCAGG) and deleted (CCTTCCCACGTTAGTGTAGGTTCTTCTC and GGAGGGTGACCACACATGAAAGTCT) alleles. This allele was confirmed to be protein null by western blotting (**Fig 1B**). Subsequent genetic experiments were performed using this null allele. A *LacZ* gene-trap allele of mouse *Ccrk* was generated from an embryonic stem cell line harboring a *LacZ* reporter inserted between exons 2 and 3 of *Ccrk* (referred to here as *Ccrk*^*tm1aLacz*^, also called *Cdk20*^*tm1a(KOMP)Wtsi*^). This line was obtained from the Knockout Mouse Project, Wellcome Trust Sanger Institute. The *Ccrk*^*tm1aLacz*^ mouse line was used to characterize the *Ccrk* expression pattern (S2 Fig). Mice homozygous for this mutation showed a phenotype very similar to the *Ccrk*^*KO*^ allele described above.

### Mouse and cell lines

#### Mouse lines

In addition to *Ccrk* mutant alleles (described above), other strains used in the study were obtained from Dr. Kathryn Anderson (*Rab23*^*opb2*^, [32]), Andrew McMahon (*Smo*^*tm1Amc*^, [48]), Alexandra Joyner (*Gli2*^*zfd*^, [33]), and Jackson Laboratories (*Gli3*^*Xt-J*^, Stock# 000026, [49]) and genotyped according the protocols in the associated references. The *bromi* allele was genotyped according our previously published protocol [23]. All alleles are functionally null and all were maintained on a C3H/HeJ background for >4 generations.

#### Cells

Primary mouse embryonic fibroblast cultures were generated from E13.5 embryos and cultured as described [50]. Immortalized MEF lines were derived from these primary cultures using established methods [50]. Complementation of the *Ccrk* null immortal lines was performed by transfecting a plasmid containing wild-type or K33R mutant *Ccrk* driven by a ubiquitous promoter (pUB6-CCRK/V5/His). Clonal lines were established and genotyped. Expression of transgenic CCRK was verified by western blot using anti-V5 antibody.

An mIMCD3 IFT88::YFP stable cell line that has been previously used to study IFT transport [16, 38] was a kind gift from Dr. Jagesh Shah (Harvard Medical School). IMCD3 Cells were cultured as described [16]. During live-imaging cells were switched to phenol red free media with 25mM HEPES.

A *Ccrk* null mutation was generated in these cells using CRISPR/Cas9 mutagenesis as described [51]. Briefly, we used bioinformatics approaches to find a candidate gRNA target site (CCCACATGGTGCGGGCTTTGTGCT) in exon 3 of mouse *Ccrk*. The sequence was BLASTed against the mouse genome to confirm it was unique with low chance of off-target mutagenesis. We generated a gBLOCK (IDT) fragment containing this target as well as the remaining guide RNA sequence from [54].

hCas9 (a gift from George Church (Addgene plasmid # 41815, [51]), the gRNA PCR product, and a puromycin resistance plasmid were transfected into stable IMCD IFT88::YFP cells to generate stable lines. Genotyping of clones identified a clone heterozygous for a 67 bp insertion (5’-CTATTCCATTAGGCTAAG TAATATGCCATTGACTATTTTAAAAATGAGA AATCTAATGGATAGAGAA-3’). Homozygous clones were generated using a second round of mutagenesis. Clones were analyzed to identify homozygote lines created through gene conversion. Mutant IMCD3 cells were complemented through stable transfection with a pUB6-CCRK/V5/His plasmid vector. Clonal stable transgenic lines were generated and verified. Complementation of the IMCD *Ccrk*^*null*^ phenotype was verified based on IFT88::YFP expression patterns.

### Mouse embryonic fibroblast culture and Hedgehog pathway manipulation

Control and *Ccrk* null MEFs were exposed to varying concentrations of Smoothened agonist (SAG, Cayman Chemical), or to fixed concentrations of SAG for varying time periods, followed by harvesting of RNA, with vehicle (DMSO) serving as a negative control. All experiments were performed in triplicate.

In hysteresis experiments, MEFs were induced with 200 nM SAG for 8 hours. The SAG was then washed out and the Smoothened inhibitor Cyclopamine (10 μM, Calbiochem), was added to counteract any signaling that could result from remaining SAG. In other samples, cells were continually exposed to SAG for up to 72 h. The relative, normalized expression of *Gli1* was determined using qPCR.

### Quantitation of relative Hh target gene expression

RNA was extracted from MEFs using the E.Z.N.A. Total RNA Kit I (Omega R6834-00). cDNA was synthesized using qScript (Quanta). RT-qPCR was performed using *Gli1* (IDT Mm.PT.58.11933824) and *Ptch1* (IDT Mm.PT.58.5305068), and normalized to *Actb* (IDT Mm.PT.58.33540333) levels using an Applied Biosystems 7500 Real Time PCR System. Normalized values were averaged among triplicates and expressed with standard error.

### Cell immunohistochemistry, microscopy and quantitation

Control and *Ccrk* mutant MEFs were grown to confluency on glass coverslips and serum starved in 1% FBS for 24 hours to induce ciliogenesis. The cells were processed for staining using standard protocols [54] with the following antibodies: rabbit polyclonal against mouse Smoothened (kindly provided by Dr. Kathryn Anderson, Sloan Kettering Institute), rabbit polyclonal against Arl13b (Kindly provided by Dr. Tamara Caspary, Emory University), mouse monoclonal against acetylated α-tubulin (Sigma-Aldrich), and a mouse monoclonal against γ-tubulin (Sigma-Aldrich). Cells were stained with appropriate secondary antibodies together with DAPI. Images were taken on Zeiss Axioplan 2 microscope with a 100x oil objective and analyzed using AxioVision4.6 and FIJI Software.

MEFs were exposed to 200 nM SAG for time periods ranging from 30 min to 24 hours and then fixed. Experiments were performed in triplicate. Cells were immunostained as described above to visualize the localization of Gli2 and Smoothened to the cilium after different lengths of exposure to SAG. Fluorescent intensity was determined using ImageJ software. Background fluorescence was used to set thresholds for measuring frequency of cilia showing localization. Values were obtained for 50–200 cilia per condition/coverslip.

Ciliary length was determined for control and *Ccrk* null MEFs through immunolabeling to visualize steady-state cilia length. The axonemes of cilia were labeled with Arl13b and acetylated α-tubulin, whereas the centrosome was labeled with γ-tubulin. Cilia in the focal plane were measured from the base of the cilium where it intersected the centrosome to the tip of the Arl13b staining. Length measurements were performed using Zeiss 4.0 and ImageJ Software.

### Embryo processing and immunostaining

E9.5–11.5 embryos were dissected and prepared for cryosectioning using standard protocols [40]. Cryosections were stained with primary antibodies and fluorescent secondary antibodies (Jackson Immunologicals), followed by counterstaining with DAPI. Primary antibodies were: mouse anti-Shh 5E1, mouse anti-Nkx2.2, mouse anti-HB9, mouse anti-Isl1/2, mouse anti-FoxA2, mouse anti-Nkx6.1, mouse anti-Pax7 (all from DSHB at 1:10), and sheep anti-Chx10 (1:600, Exalpha), and rabbit anti-Pax6 (1:300, Covance).

### Transmission and scanning electron microscopy

Wild-type and *Ccrk* homozygous null IMCD3 cells were grown to confluency, followed by fixation in 2% glutaraldehyde/0.01% tannic acid and post-fixation in 1% O_4_Os. Samples were embedded in EPON resin for Transmission Electron Microscopy (TEM) or gold-coated (Structure Probe, Inc) for Scanning Electron Microscopy (SEM). Sections for TEM were cut using a RMC MT-X microtome (RMC Products) and placed on copper grids and visualized using a JEOL JEM-1210 Transmission Electron Microscope (JEOL, Inc.). SEM samples were visualized using a Zeiss 1450EP SEM microscope (Carl Zeiss MicroImaging).

### Measurement of IFT parameters

Live imaging was performed using a Total Internal Reflection Fluorescence (TIRF) microscope [52] at 60X magnification with videos taken at 10 frames per second. Cells were plated on inverted Transwell® membranes (Corning) and grown for 7 days as described [53]. The videos were analyzed by generating kymographs using ImageJ. Anterograde and retrograde IFT speeds and frequencies were calculated by taking measurements of the IFT88::YFP tracks seen on the kymographs [52].

### In situ hybridization, skeletal analysis, and β-galactosidase staining

*In situ* hybridization was performed on frozen sections from E9.5 embryos according to methods previously described [54]. Whole-mount in situ hybridization was performed according to methods previously described [51]. Riboprobes were synthesized using a kit from Roche according to the manufacturer’s instructions from the following plasmid templates: *Shh* [55], *Gli1* [56], *Olig2* [31], and *Pax6* [57]. Analysis of *Ccrk* expression was performed using sense and antisense riboprobes synthesized from a 507 bp PCR-amplified fragment of the 3’ UTR region of mouse *Ccrk* (from positions 1169–1676 in mRNA sequence ID: NM_053180.2) subcloned into pGEM-T Easy (Promega). The *Ccrk* plasmid was digested with NcoI and transcribed using the Sp6 promoter to generate sense probes or digested with SacI and transcribed using the T7 promoter to generate antisense probe.

Cartilage and bone from E16.5 *Ccrk* null and wild-type control embryos were stained with Alcian Blue and Alizarin Red according to standard protocols [47]. Whole-mount β-galactosidase staining of e10.5 *Ccrk*^*tm1aLacZ/+*^ and control embryos was performed using a standard protocol [51].

### Western blotting

Whole embryo protein lysates for immunoblotting were obtained by homogenizing E10.5 embryos in modified RIPA buffer with protease inhibitor cocktail (Roche). Protein concentration was determined by the BCA Protein Assay (Pierce). Equal amounts of protein (10 μg) were loaded for electrophoresis. After electrophoresis, proteins were transferred to PVDF membranes (Millipore). Membranes were washed with 1x TBS containing 0.2% Tween 20 (TBST) and blocked for 1 hour in TBST containing 5% skim milk. Membranes were then incubated overnight at 4°C with a mouse monoclonal anti-Gli3 (kindly provided by Dr. Susie Scales, Genentech, CA), a guinea pig polyclonal antibody against mouse Gli2 (described in [58]), a rabbit polyclonal antibody against CCRK (kindly provided by Dr. Robert Fisher, Mount Sinai Hospital, NY, NY) or mouse anti-β actin (Cell Signaling Technology). After washing, membranes were incubated with HRP-labeled secondary antibodies. Signals were detected with a chemiluminescent reagent (Millipore) following the manufacturer’s instructions. Signals were quantitated using ImageJ.

### In vitro phosphatase treatment

Whole embryo protein lysates for *in vitro* phosphatase treatment were prepared from e9.5 embryos by clarifying in Triton lysis buffer (1% triton X-100, 50mM HEPES, 150mM NaCl, 2mM DTT) with protease inhibitor cocktail (Roche) for 15 min at 17,000 × g at 4°C. Thirty micrograms of extracts plus lambda phosphatase reaction buffer supplied with manufacturer (NEB, MA) in 50 ul total reaction volume were incubated at 30°C for 30 min with 400 unit lambda phosphatase. After phosphatase treatment, the reactions were stopped and run on SDS-PAGE, followed by western blotting analysis with a guinea pig polyclonal anti-Gli2 antibody.

### Luciferase assays

Wild-type and *Ccrk*^*KO/KO*^ pMEFs were cultured as described above. Cells were transfected using Lipofectamine 2000 reagent (Invitrogen) with the 8xGliBS-Luc [59], containing eight copies of Gli-binding elements fused to the firely *luciferase* gene, as well as a Renilla luciferase construct for normalization. Cells were serum starved in OptiMEM (GIBCO) for 24 h and treated for an additional 24 h period with varying concentrations of SAG or DMSO (control) in serum-free media. Gli reporter activity was assayed using the Dual-Luciferase® Reporter Assay System (Promega). Firefly luciferase activity was normalized to Renilla luciferase activity. Experiments were performed in triplicate.

### Quantitation and statistical analysis

Quantitation of cells positive for markers in the neural tube was determined by counting positive cells per section from two sections per embryo with an average obtained. The positions of dorsal and ventral limits of expression domains were determined by measuring the total ventral-dorsal length of the neural tube in sections and expressing the positions of these expression domain limits as fractions of the total ventral-to-dorsal length. Values were analyzed across 3 embryos per genotype per stage to obtain overall averages, standard deviations, and statistical significance. Statistical analysis was conducted using either Student’s t-tests or Chi-square tests (for frequencies of Gli2 and Smo ciliary localization), computed using StatPlus software (Analystsoft).

## Supporting information

S1 Fig Cleft palate phenotype in *Ccrk* and *Bromi* mutants. E17.5 wild-type (a,d,g), *Ccrk* mutant (b,e,h) and *Bromi* mutant (c,f,i) dissected heads show cleft palate (arrows in e and f) in both mutants. Histological staining of sections (g,h,i) reveals fusion defects of palatal shelves in the mutants (arrowheads in h,i).
(TIF)

S2 Fig The *Ccrk* expression pattern at E10.5. (A) *Ccrk* expression was assayed using by in situ hybridization using an antisense probe directed against the 3’ untranslated region of the gene. *Ccrk* expression in E10.5 wild-type embryos appeared weak and mostly uniform throughout the embryo with slightly more intense staining in the limb buds and pharyngeal arches and weak-to-no staining in the heart. This staining appeared to be specific because staining of *Ccrk* null homozygotes with the antisense probe (as well as staining of wild-type embryos with a *Ccrk* sense probe) showed no clear signal. (B) The uniform expression pattern was confirmed by β-galactosidase staining of embryos heterozygous for a *LacZ* gene-trap insertion in *Ccrk* (*Ccrk*^*tm1aLacZ*^).
(TIF)

S3 Fig *In situ* hybridization analysis of E9.5 neural tube patterning. *Shh* expression (a,b) was seen in the *Ccrk* mutant notochord (b) but not in the ventral neural tube. *Gli1* (c,d) staining intensity was reduced in *Ccrk* mutant neural tube sections (d). *Olig2* (e,f) was expressed in a domain that expanded dorsally and ventrally (across the ventral midline) in *Ccrk* mutants (f) relative to controls (e). *Pax6* (g,h) expression was observed in ectopic ventral domains in the mutant neural tube (h).
(TIF)

S4 Fig Quantitation of Shh-dependent neural tube patterning as a function of developmental stage in *Ccrk* mutants. Wild-type and *Ccrk* mutant embryos were obtained between E9.0 and E11.5 and somite number determined. Sections at the 4-5^th^ somite level were stained for FoxA2, Nkx2.2, Olig2, and Pax6. Numbers of expressing cells (FoxA2, Nkx2.2, Olig2) as well as ventrally-positioned nonexpressing cells (Pax6) were counted. As early as the 10-13-somite stage, *Ccrk* mutants showed a dorsalized pattern manifested as fewer Fox2+, Nkx2.2+, Olig2+, and Pax6- cells. By the 24-27-somite stage, the Olig2+ domain had expanded in the mutant. Vnt, ventral neural tube. Quantitation derived from 3 embryos per genotype/stage (2 sections per embryo). Error bars represent standard error of the mean. P values from Student’s t-tests: *, p<0.05; **, p<0.01; ns, not significant.
(TIF)

S5 Fig *Ccrk* is epistatic to *Rab23* with respect to neural tube patterning. Sections through the lumbar neural tubes of E10.5 wild-type (a-e), *Ccrk* single mutant (f-j), *Rab23* single mutant (k-o), and *Ccrk/Rab23* double mutants (p-t) were stained for Shh (a,f,k,p), FoxA2 (b,g,l,q), Nkx2.2 (c,h,m,r), HB9 (d,I,n,s), and Pax6 (e,j,o,t). Whereas ventral markers (Shh, FoxA2, Nkx2.2) showed a dorsally expanded profile in *Rab23* mutants, these markers were reduced and expressed in more ventrally restricted domains in *Ccrk* mutants. Pax6 expression was inhibited in *Rab23* mutants and was ventrally expanded in *Ccrk* mutants. *Ccrk/Rab23* double mutants showed patterns indistinguishable from *Ccrk* single mutants. Results from quantitation of data from 3 embryos/genotype and statistical analysis are presented in **S2 Table**.
(TIF)

S6 Fig The *Ccrk* mutation partially suppresses the *Smoothened* mutant neural patterning phenotype. Wild-type (a-d), *Ccrk* mutant (e-h), *Smoothened* (*Smo*) mutant (i-l), and *Ccrk/Smo* double mutants (m-p) were harvested at E9.5. Morphologically, *Ccrk/Smo* double mutants resemble *Smo* single mutants (i), except that the head size was partially rescued in the double mutants (m). Sections through the rostral spinal neural tubes were stained for Nkx2.2 (b,f,j,n), Olig2 (c,g,k,o), and Nkx6.1 (d,h,l,p). Nkx2.2 expression was not rescued in the double mutants but some Olig2+ (o) and Nkx6.1+ (p) cell fates were restored. Results from quantitation of data from 3 embryos/genotype and statistical analysis are presented in **S3 Table**.
(TIF)

S7 Fig Disruption of Gli2 exacerbates the dorsalized phenotype of *Ccrk* mutant neural tube. Sections through the brachial spinal neural tubes of E11.5 wild-type (a-c), *Ccrk* single mutants (d-f), *Gli2* singe mutants (g-i), and *Ccrk/Gli2* double mutants (j-l). Note that the double mutant neural tube lacks Nkx2.2 and Shh expression (k and j, respectively), shows significant reduction of Isl1/2+ (l, in green) and Olig2+ (k, in red) motor neurons and MN progenitors, and that Chx10+ V2 interneurons (l, in red) are ectopically positioned in ventral domains in the double mutant. Quantitation of data from 3 embryos/genotype and statistical analysis of data are presented in **S4 Table**.
(TIF)

S8 Fig Loss of *Gli3* suppresses the *Ccrk* mutant neural patterning phenotype. Sections through the brachial neural tubes of E11.5 wild-type (a-d), *Gli3* null mutants (e-h), *Ccrk* mutants (i-l), *Ccrk/Gli3-/-* mutants (m-p) and *Ccrk/Gli3+/-* (q-t) mutants were stained for Shh (a,e,I,m,q), Nkx2.2 (b,f,j,n,r), Olig2 (c,g,k,o,s), and Isl1/2 (d,h,l,p.t). Ventral regions of the neural tubes are shown. Whereas the *Gli3-/-* mutants showed nearly normal patterning phenotype, the *Ccrk* mutant neural tube was partially dorsalized, as evidenced by the loss/reduction of Shh (i) and Nkx2.2 (j) staining. In *Ccrk-/-Gli3-/-* double mutants, the Shh+ floor plate was restored (m) and Nkx2.2 expression was extinguished in the ventral midline (n). *Ccrk-/-/Gli3+/-* mutants showed a variable rescue of Shh+ floor plate specification (q, n = 3/5). Results from quantitation of data from ≥3 embryos/genotype and statistical analysis are presented in **S5 Table**.
(TIF)

S9 Fig Hh pathway responses of *Ccrk* mutant MEFs over time. (A) Normalized qPCR analysis of *Gli1* expression by wild-type and *Ccrk* mutant MEFs in response to 20 nM or 5 nM Smoothened agonist (SAG) as a function of time of exposure. *Ccrk* mutant cells showed a clear deficiency in their responses at late time points. (B) Normalized qPCR analysis of *Patched1* (*Ptch1*) expression in response to 200 nM SAG over time. Although *Ccrk* mutant MEFs showed a slight deficiency in response to SAG for short periods, the defect was far more pronounced at longer periods of exposure (≥ 12 hours). Quantitation was performed using 3 biological replicates per condition. Error bars represent standard error of the mean. P values from Student’s t-tests: **, p<0.01; *, p<0.05; ns, not significant.
(TIF)

S10 Fig *Ccrk* mutant MEFs retain hysteretic activity upon removal of SAG. We treated control (*Ccrk*+/-) and *Ccrk* mutant MEFS under a series of conditions outlined in (A). Cells were treated with vehicle alone, treated continuously with 200 nM SAG for 8, 24, 58, or 72 h, or they were first treated with 200 nM SAG for 8 h (phase I) followed by SAG washout and addition of 10 μm Cyclopamine (Cyclop.) added for an additional 24, 48 or 72 h (Phase II). A diagram depicting expectations of hysteretic and non-hysteretic activity profiles after inducer removal is shown in (B). (C) Normalized qPCR analysis of *Gli1* expression under the conditions outlined in A. *Ccrk* mutant MEFs mounted normal responses to SAG after 8 hours of exposure and they retained levels of *Gli1* expression comparable to controls even after signaling had been terminated for 24–72 h. Quantitation was performed using 3 biological replicates per condition. Error bars represent standard error of the mean. P values from Student’s t-tests: **, p<0.01; *, p<0.05; ns, not significant.
(TIF)

S11 Fig Immunofluorescent analysis of cilia in the spinal neural tubes of wild-type and *Ccrk* mutant embryos. (A) Low magnification confocal images of the ventral neural tubes of E10.5 wild-type and *Ccrk* mutants stained for Arl13b (red) and DAPI (blue). Note that cilia were abundantly generated in the mutants. (B) High magnification images of neural tube cilia stained for γ-tubulin (red) to highlight basal bodies and Arl13b (green) to highlight cilia. Scale bars are 10 and 4 μm in A and B, respectively.
(TIF)

S12 Fig Model depicting the function of CCRK in ciliary cargo transport and the implications for activity of the Hh signaling pathway. (A) We propose that CCRK acts through an unknown mechanism to promote the loading of ciliary cargo onto IFT particles so that they may be imported into the cilium. Such cargo includes regulators of Hh signal transduction (e.g. Gli2), tubulin dimers, and potentially, regulators of ciliary assembly/disassembly. (B) An individual Gli transcription factor molecule must first be loaded onto transport machinery and then transported towards the cilium tip where it can be activated (via its dissociation from Sufu). Once activated, the Gli molecule must be transported back to the cell body where it can enter the nucleus and activate target genes. We suggest that the initial step (loading and transport) is inefficient in *Ccrk* mutant cells. This would have the effect of delaying the time required for an individual molecule to be imported, activated, and function. Hence, fewer activated Gli molecules would be generated in the *Ccrk* mutant per unit of time. (C) During the period of Hh pathway induction, normal cells show two phases: an initial phase of slow increase, during which *Gli2* is upregulated (due to feedback), and a second phase when the magnitude of the response is amplified (possibly due to the increased levels of Gli2). We suggest that the initial phase is relatively insensitive to the efficiency of ciliary import of Hh pathway regulators, but the transport efficiency is rate limiting for the response during the amplification phase. Thus, the decrease in the rate of ciliary transport of Hh pathway regulators in *Ccrk* mutant cells has very little effect during the initial phase, but it has a dramatic effect on the response during the amplification phase.
(TIF)

S1 Table Quantitation of patterning changes in E10.5 neural tubes (Fig 2). Immunostained sections from embryos of Wild-type and *Ccrk-/-* genotypes were analyzed with respect to a) the position of dorsal and ventral limits of expression domains as a fraction of neural tube size (Pax6, Isl1/2, HB9) or b) numbers of marker positive cells per section (Nkx2.2 and Shh). Numbers of embryos per genotype and numbers of sections per embryo analyzed are shown, in addition to average values, standard deviations, and P values from Student’s t-tests.
(TIF)

S2 Table Quantitation of patterning changes in Ccrk/Rab23 experiment (S5 Fig). Immunostained sections from embryos of Wild-type, *Ccrk-/-*, *Rab23-/-*, and *Ccrk-/-Rab23-/-* genotypes were analyzed with respect to numbers of FoxA2+, Shh+, and Nkx2.2+ cells per neural tube section. Numbers of embryos per genotype and numbers of sections per embryo analyzed are shown, in addition to average values, standard deviations, and P values from Student’s t-tests.
(TIF)

S3 Table Quantitation of patterning changes in *Ccrk/Smo* experiment (S6 Fig). Immunostained sections from embryos of Wild-type, *Ccrk-/-*, *Smo-/-*, and *Ccrk-/-Smo-/-* genotypes were analyzed with respect to numbers of Nkx2.2+, Olig2+, and Nkx6.1+ cells per neural tube section. Numbers of embryos per genotype and numbers of sections per embryo analyzed are shown, in addition to average values, standard deviations, and P values from Student’s t-tests.
(TIF)

S4 Table Quantitation of patterning changes in *Ccrk/Gli2* experiment (S7 Fig). Immunostained sections from embryos of Wild-type, *Ccrk-/-*, *Gli2-/-*, and *Ccrk-/-Gli2-/-* genotypes were analyzed with respect to numbers of Nkx2.2+, Olig2+, and Isl1/2+ cells per neural tube section. Numbers of embryos per genotype and numbers of sections per embryo analyzed are shown, in addition to average values, standard deviations, and P values from Student’s t-tests.
(TIF)

S5 Table Quantitation of patterning changes in *Ccrk/Gli3* experiment (S8 Fig). Immunostained sections from embryos of Wild-type, *Ccrk-/-*, *Gli3-/-*, *Ccrk-/-Gli3+/-* and *Ccrk-/-Gli3-/-* genotypes were analyzed with respect to numbers of Shh+ cells per neural tube section. Numbers of embryos per genotype and numbers of sections per embryo analyzed are shown, in addition to average values, standard deviations, and P values from Student’s t-tests.
(TIF)
