# Supplementary figures and images for: Cell Cycle-Related Kinase (CCRK) regulates ciliogenesis and Hedgehog signaling in mice
Source: PLoS Genet. 2017 Aug 17;13(8):e1006912. doi: 10.1371/journal.pgen.1006912 (PMC5574612; doi:10.1371/journal.pgen.1006912)

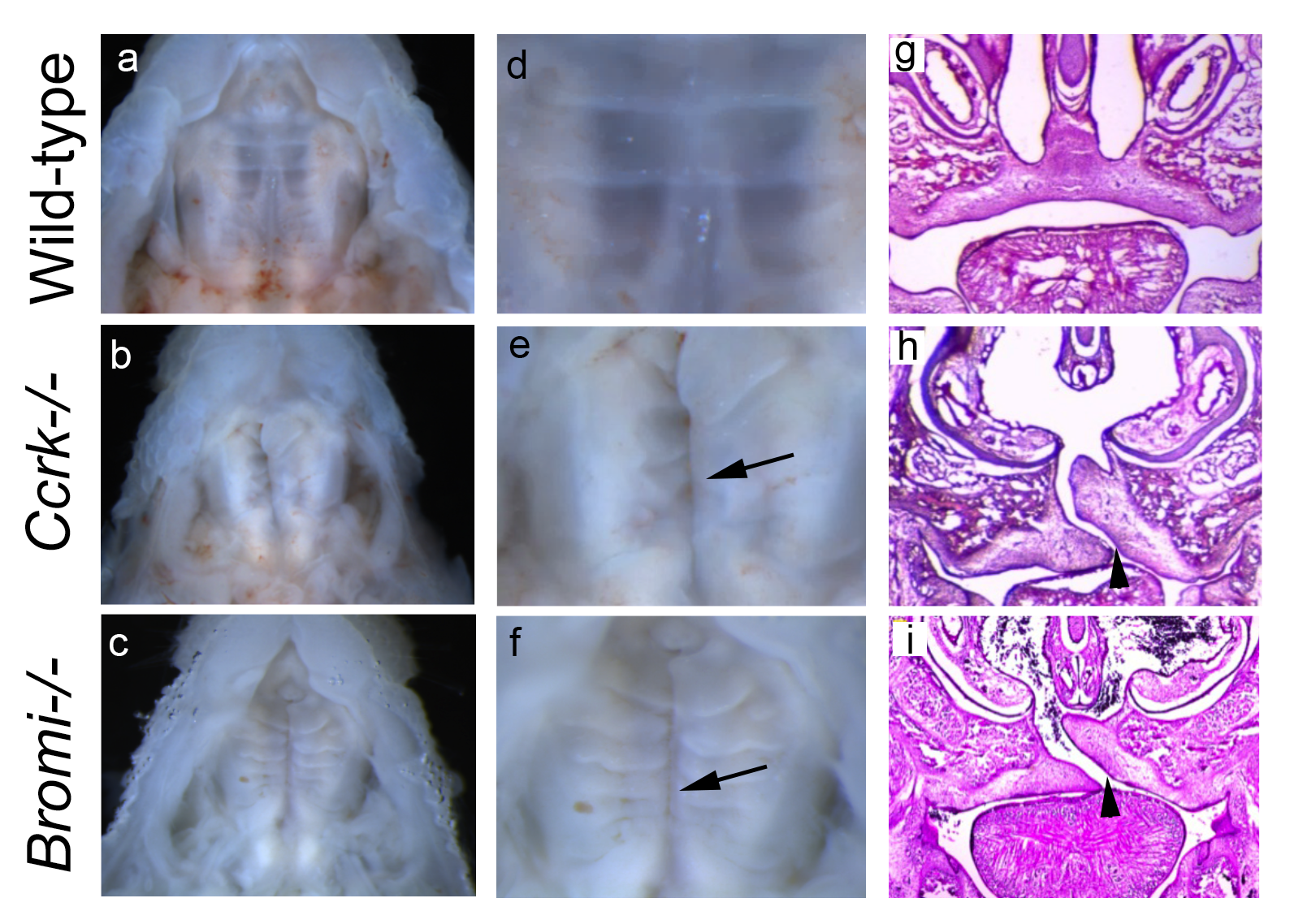

Supplement: S1 Fig — E17.5 wild-type (a,d,g), Ccrk mutant (b,e,h) and Bromi mutant (c,f,i) dissected heads show cleft palate (arrows in e and f) in both mutants. Histological staining of sections (g,h,i) reveals fusion defects of palatal shelves in the mutants (arrowheads in h,i). (TIF) [file pgen.1006912.s001.tif]

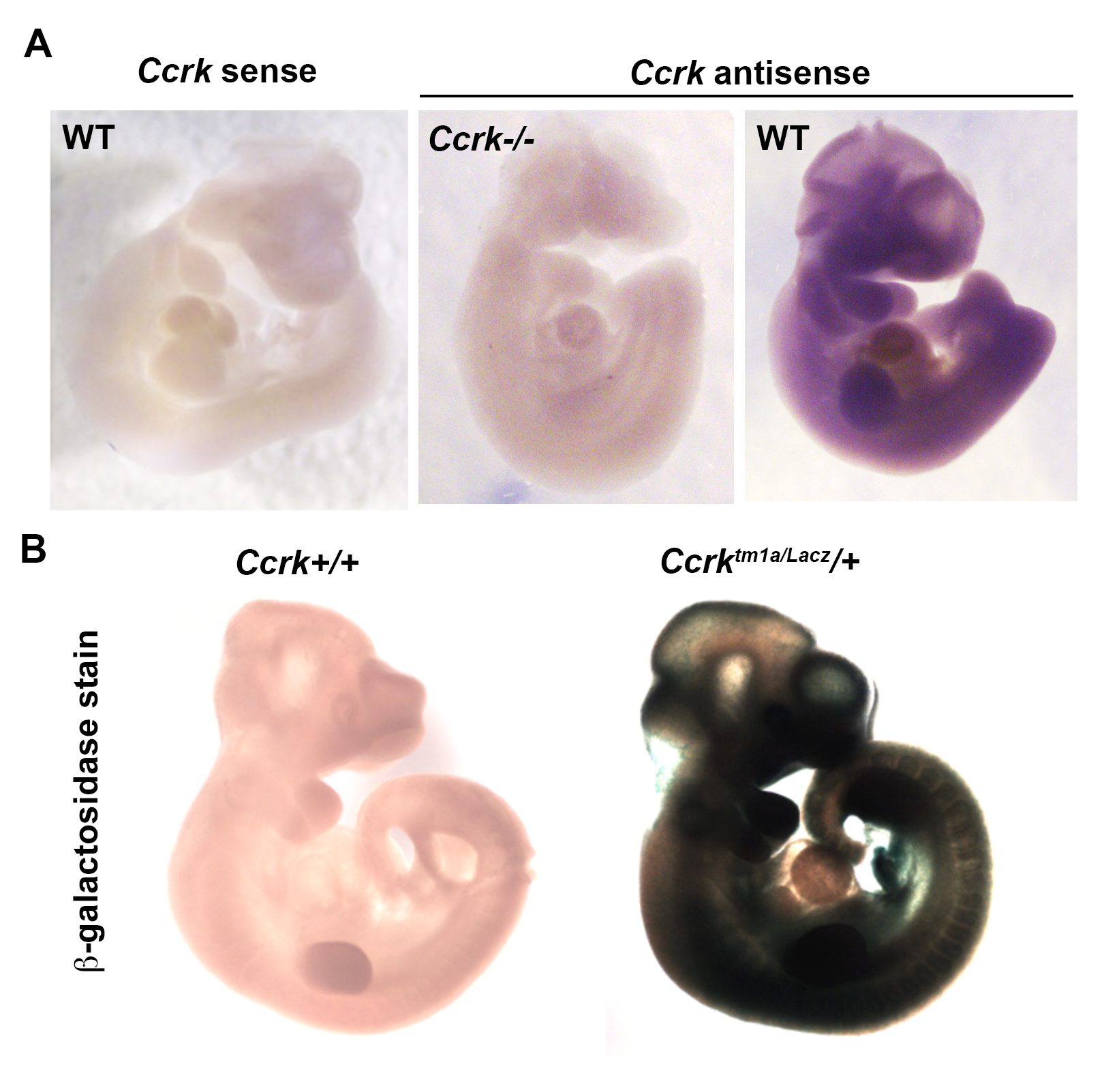

Supplement: S2 Fig — (A) Ccrk expression was assayed using by in situ hybridization using an antisense probe directed against the 3’ untranslated region of the gene. Ccrk expression in E10.5 wild-type embryos appeared weak and mostly uniform throughout the embryo with slightly more intense staining in the limb buds and pharyngeal arches and weak-to-no staining in the heart. This staining appeared to be specific because staining of Ccrk null homozygotes with the antisense probe (as well as staining of wild-type embryos with a Ccrk sense probe) showed no clear signal. (B) The uniform expression pattern was confirmed by β-galactosidase staining of embryos heterozygous for a LacZ gene-trap insertion in Ccrk (Ccrktm1aLacZ). (TIF) [file pgen.1006912.s002.tif]

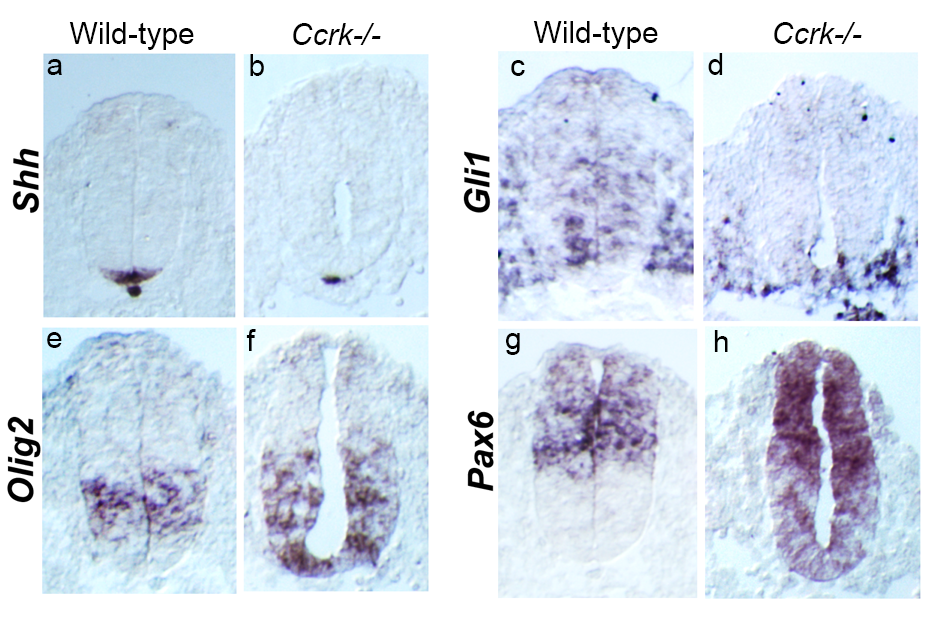

Supplement: S3 Fig — Shh expression (a,b) was seen in the Ccrk mutant notochord (b) but not in the ventral neural tube. Gli1 (c,d) staining intensity was reduced in Ccrk mutant neural tube sections (d). Olig2 (e,f) was expressed in a domain that expanded dorsally and ventrally (across the ventral midline) in Ccrk mutants (f) relative to controls (e). Pax6 (g,h) expression was observed in ectopic ventral domains in the mutant neural tube (h). (TIF) [file pgen.1006912.s003.tif]

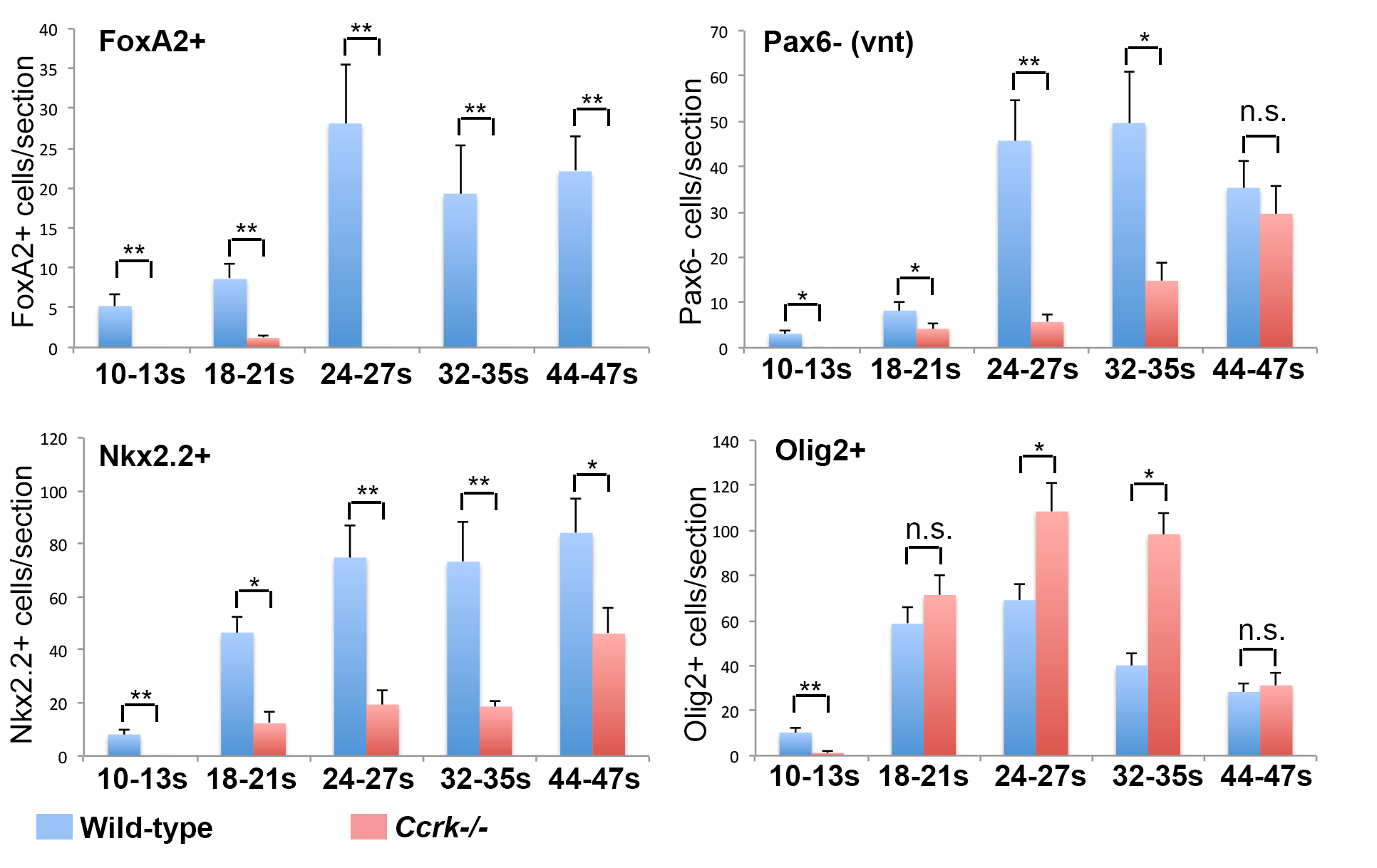

Supplement: S4 Fig — Wild-type and Ccrk mutant embryos were obtained between E9.0 and E11.5 and somite number determined. Sections at the 4-5th somite level were stained for FoxA2, Nkx2.2, Olig2, and Pax6. Numbers of expressing cells (FoxA2, Nkx2.2, Olig2) as well as ventrally-positioned nonexpressing cells (Pax6) were counted. As early as the 10-13-somite stage, Ccrk mutants showed a dorsalized pattern manifested as fewer Fox2+, Nkx2.2+, Olig2+, and Pax6- cells. By the 24-27-somite stage, the Olig2+ domain had expanded in the mutant. Vnt, ventral neural tube. Quantitation derived from 3 embryos per genotype/stage (2 sections per embryo). Error bars represent standard error of the mean. P values from Student’s t-tests: *, p<0.05; **, p<0.01; ns, not significant. (TIF) [file pgen.1006912.s004.tif]

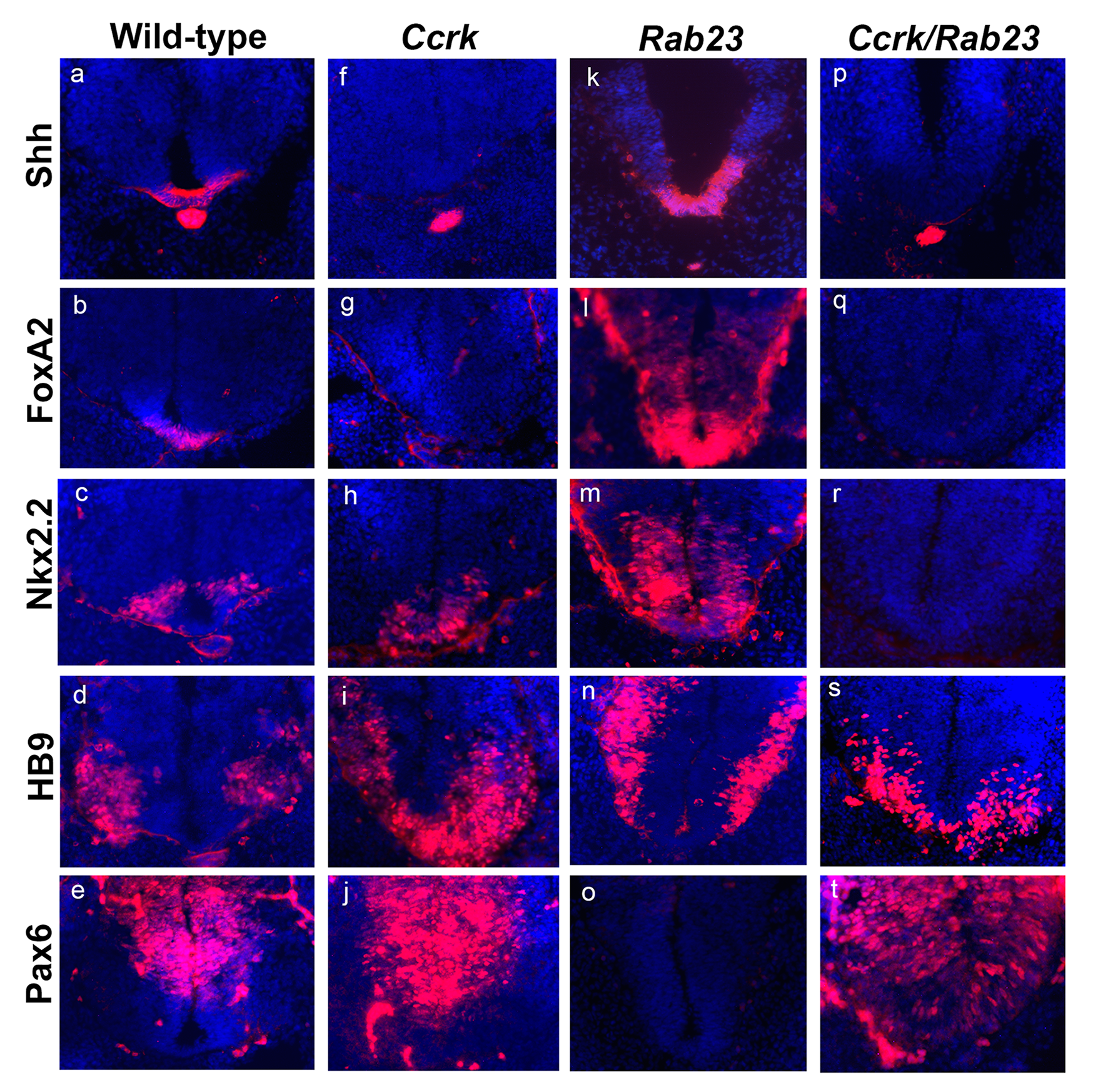

Supplement: S5 Fig — Sections through the lumbar neural tubes of E10.5 wild-type (a-e), Ccrk single mutant (f-j), Rab23 single mutant (k-o), and Ccrk/Rab23 double mutants (p-t) were stained for Shh (a,f,k,p), FoxA2 (b,g,l,q), Nkx2.2 (c,h,m,r), HB9 (d,I,n,s), and Pax6 (e,j,o,t). Whereas ventral markers (Shh, FoxA2, Nkx2.2) showed a dorsally expanded profile in Rab23 mutants, these markers were reduced and expressed in more ventrally restricted domains in Ccrk mutants. Pax6 expression was inhibited in Rab23 mutants and was ventrally expanded in Ccrk mutants. Ccrk/Rab23 double mutants showed patterns indistinguishable from Ccrk single mutants. Results from quantitation of data from 3 embryos/genotype and statistical analysis are presented in S2 Table. (TIF) [file pgen.1006912.s005.tif]

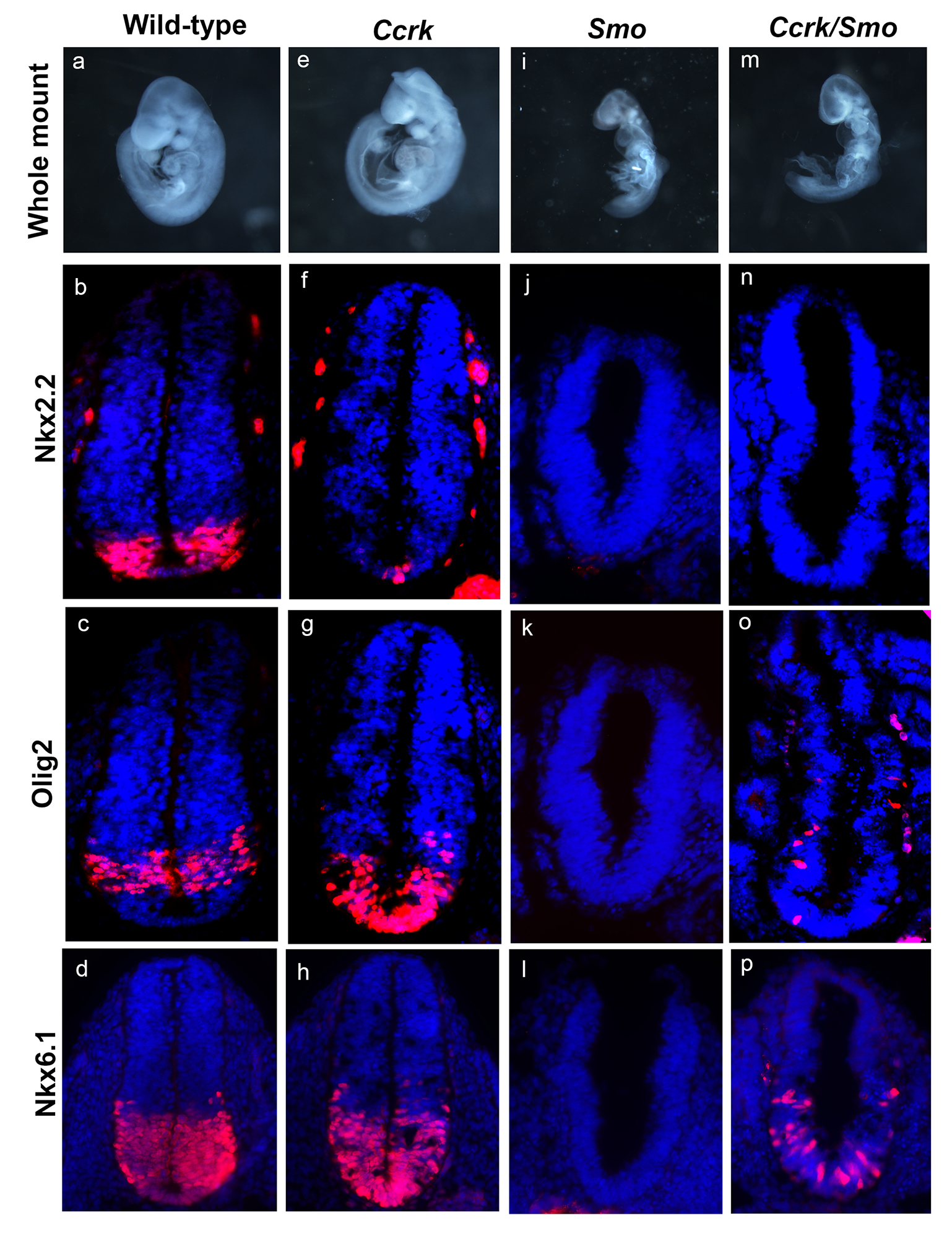

Supplement: S6 Fig — Wild-type (a-d), Ccrk mutant (e-h), Smoothened (Smo) mutant (i-l), and Ccrk/Smo double mutants (m-p) were harvested at E9.5. Morphologically, Ccrk/Smo double mutants resemble Smo single mutants (i), except that the head size was partially rescued in the double mutants (m). Sections through the rostral spinal neural tubes were stained for Nkx2.2 (b,f,j,n), Olig2 (c,g,k,o), and Nkx6.1 (d,h,l,p). Nkx2.2 expression was not rescued in the double mutants but some Olig2+ (o) and Nkx6.1+ (p) cell fates were restored. Results from quantitation of data from 3 embryos/genotype and statistical analysis are presented in S3 Table. (TIF) [file pgen.1006912.s006.tif]

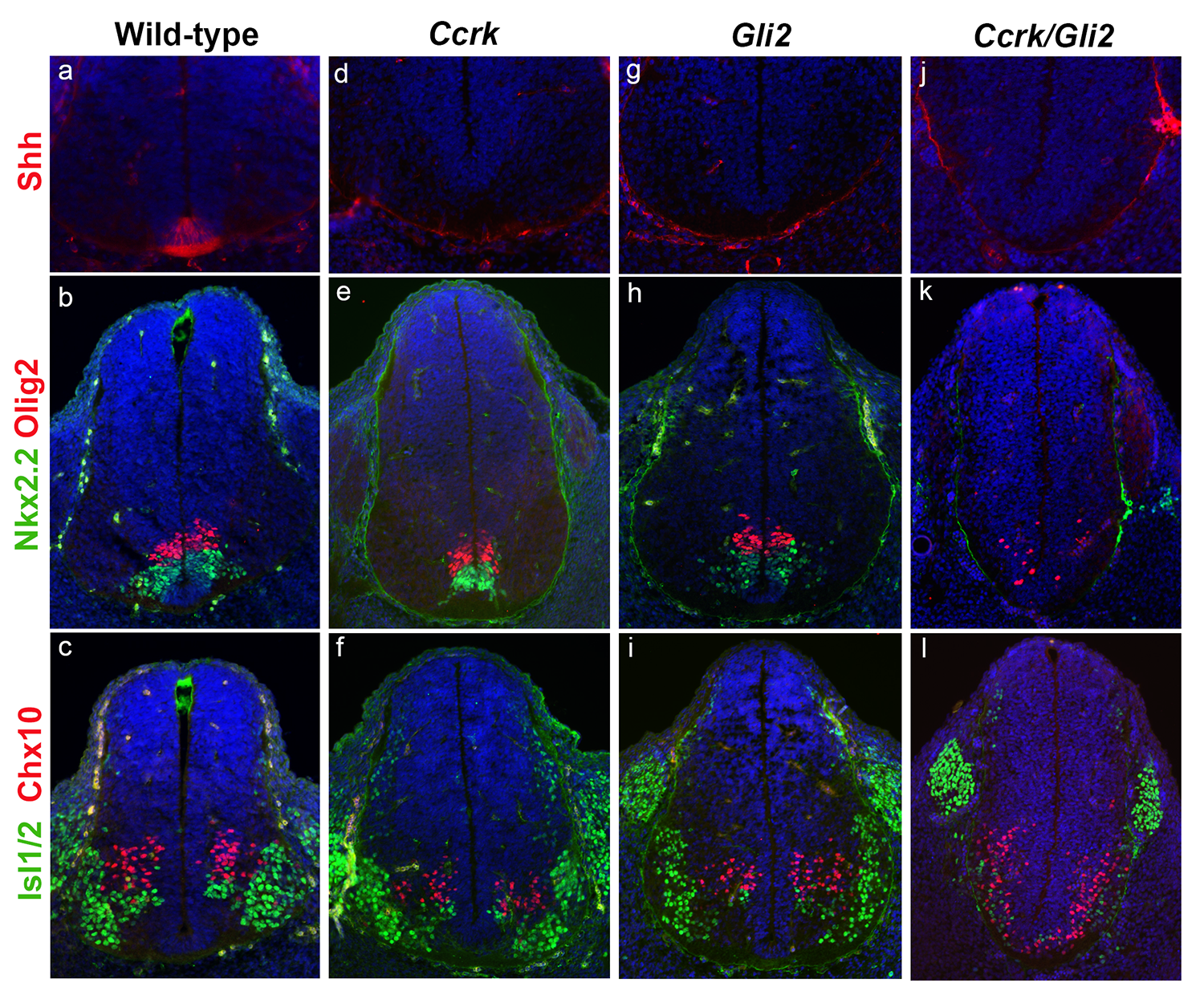

Supplement: S7 Fig — Sections through the brachial spinal neural tubes of E11.5 wild-type (a-c), Ccrk single mutants (d-f), Gli2 singe mutants (g-i), and Ccrk/Gli2 double mutants (j-l). Note that the double mutant neural tube lacks Nkx2.2 and Shh expression (k and j, respectively), shows significant reduction of Isl1/2+ (l, in green) and Olig2+ (k, in red) motor neurons and MN progenitors, and that Chx10+ V2 interneurons (l, in red) are ectopically positioned in ventral domains in the double mutant. Quantitation of data from 3 embryos/genotype and statistical analysis of data are presented in S4 Table. (TIF) [file pgen.1006912.s007.tif]

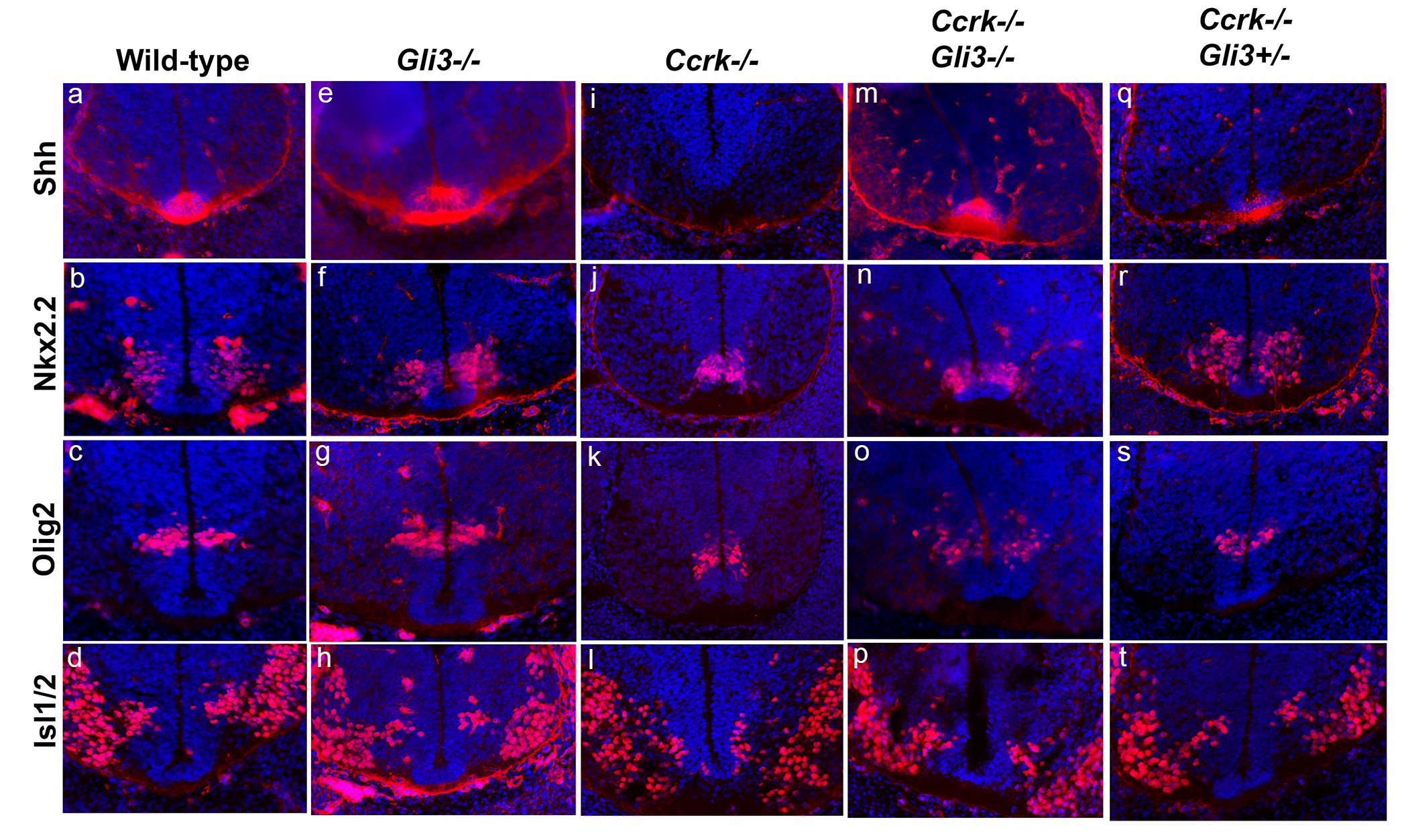

Supplement: S8 Fig — Sections through the brachial neural tubes of E11.5 wild-type (a-d), Gli3 null mutants (e-h), Ccrk mutants (i-l), Ccrk/Gli3-/- mutants (m-p) and Ccrk/Gli3+/- (q-t) mutants were stained for Shh (a,e,I,m,q), Nkx2.2 (b,f,j,n,r), Olig2 (c,g,k,o,s), and Isl1/2 (d,h,l,p.t). Ventral regions of the neural tubes are shown. Whereas the Gli3-/- mutants showed nearly normal patterning phenotype, the Ccrk mutant neural tube was partially dorsalized, as evidenced by the loss/reduction of Shh (i) and Nkx2.2 (j) staining. In Ccrk-/-Gli3-/- double mutants, the Shh+ floor plate was restored (m) and Nkx2.2 expression was extinguished in the ventral midline (n). Ccrk-/-/Gli3+/- mutants showed a variable rescue of Shh+ floor plate specification (q, n = 3/5). Results from quantitation of data from ≥3 embryos/genotype and statistical analysis are presented in S5 Table. (TIF) [file pgen.1006912.s008.tif]

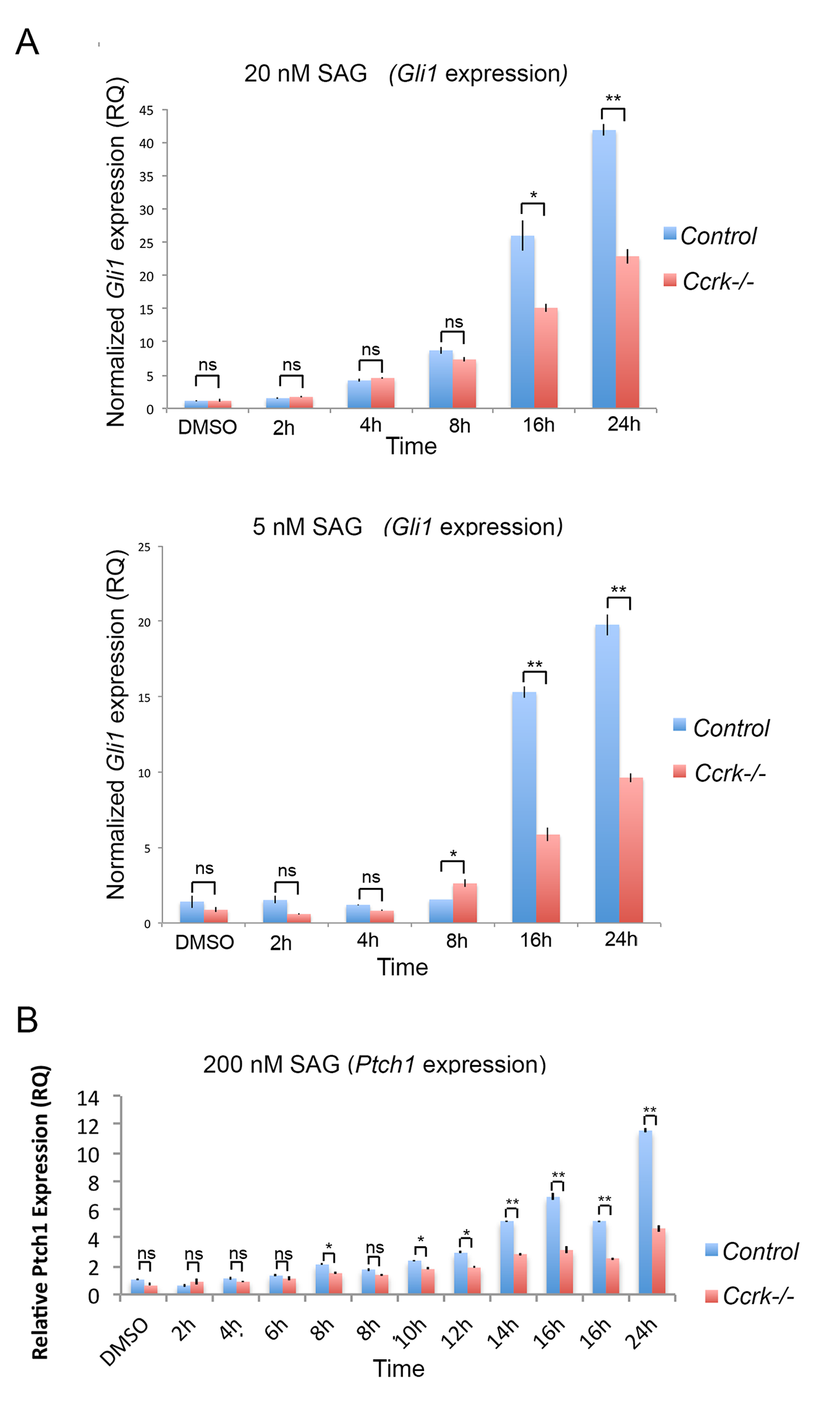

Supplement: S9 Fig — (A) Normalized qPCR analysis of Gli1 expression by wild-type and Ccrk mutant MEFs in response to 20 nM or 5 nM Smoothened agonist (SAG) as a function of time of exposure. Ccrk mutant cells showed a clear deficiency in their responses at late time points. (B) Normalized qPCR analysis of Patched1 (Ptch1) expression in response to 200 nM SAG over time. Although Ccrk mutant MEFs showed a slight deficiency in response to SAG for short periods, the defect was far more pronounced at longer periods of exposure (≥ 12 hours). Quantitation was performed using 3 biological replicates per condition. Error bars represent standard error of the mean. P values from Student’s t-tests: **, p<0.01; *, p<0.05; ns, not significant. (TIF) [file pgen.1006912.s009.tif]

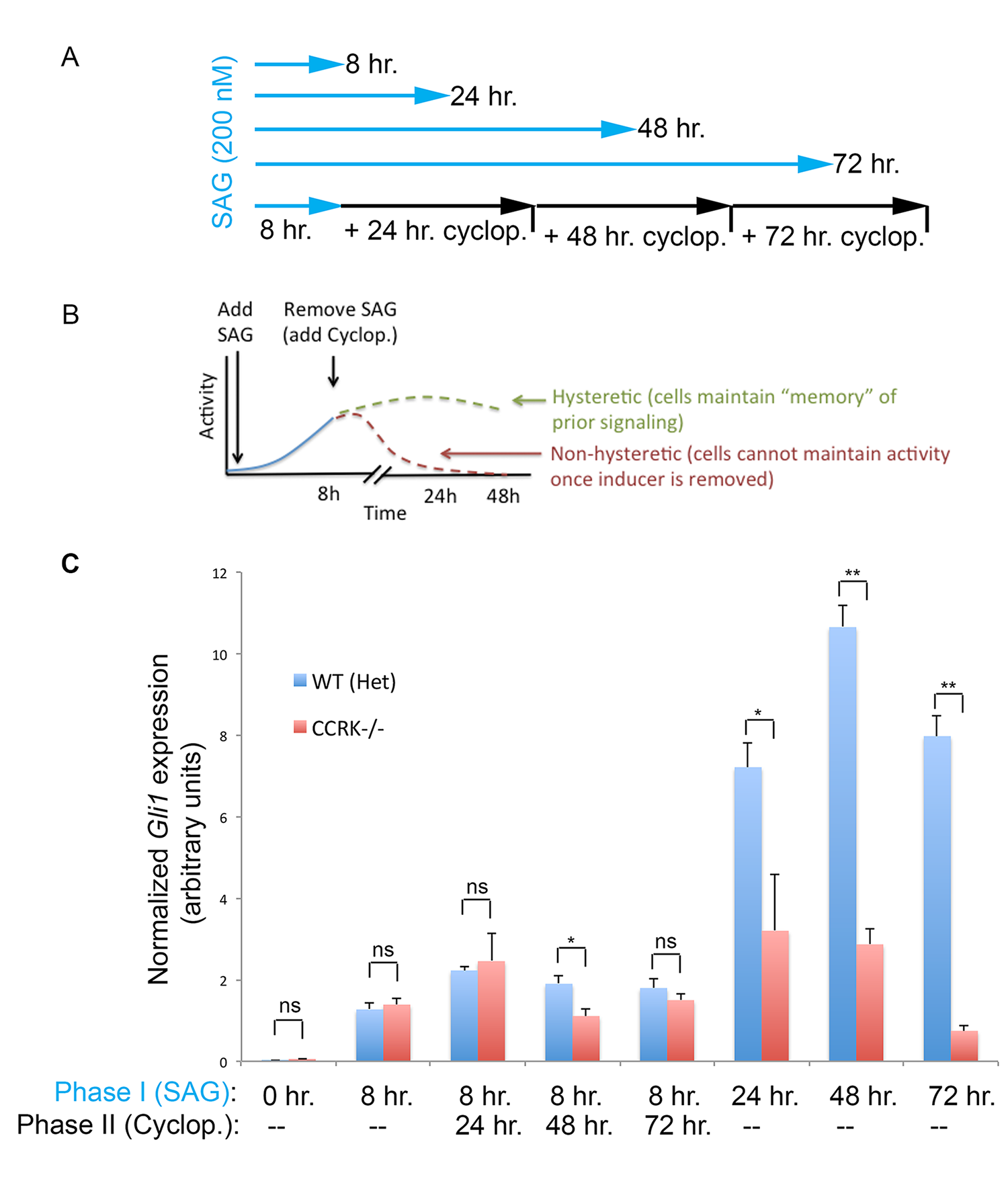

Supplement: S10 Fig — We treated control (Ccrk+/-) and Ccrk mutant MEFS under a series of conditions outlined in (A). Cells were treated with vehicle alone, treated continuously with 200 nM SAG for 8, 24, 58, or 72 h, or they were first treated with 200 nM SAG for 8 h (phase I) followed by SAG washout and addition of 10 μm Cyclopamine (Cyclop.) added for an additional 24, 48 or 72 h (Phase II). A diagram depicting expectations of hysteretic and non-hysteretic activity profiles after inducer removal is shown in (B). (C) Normalized qPCR analysis of Gli1 expression under the conditions outlined in A. Ccrk mutant MEFs mounted normal responses to SAG after 8 hours of exposure and they retained levels of Gli1 expression comparable to controls even after signaling had been terminated for 24–72 h. Quantitation was performed using 3 biological replicates per condition. Error bars represent standard error of the mean. P values from Student’s t-tests: **, p<0.01; *, p<0.05; ns, not significant. (TIF) [file pgen.1006912.s010.tif]

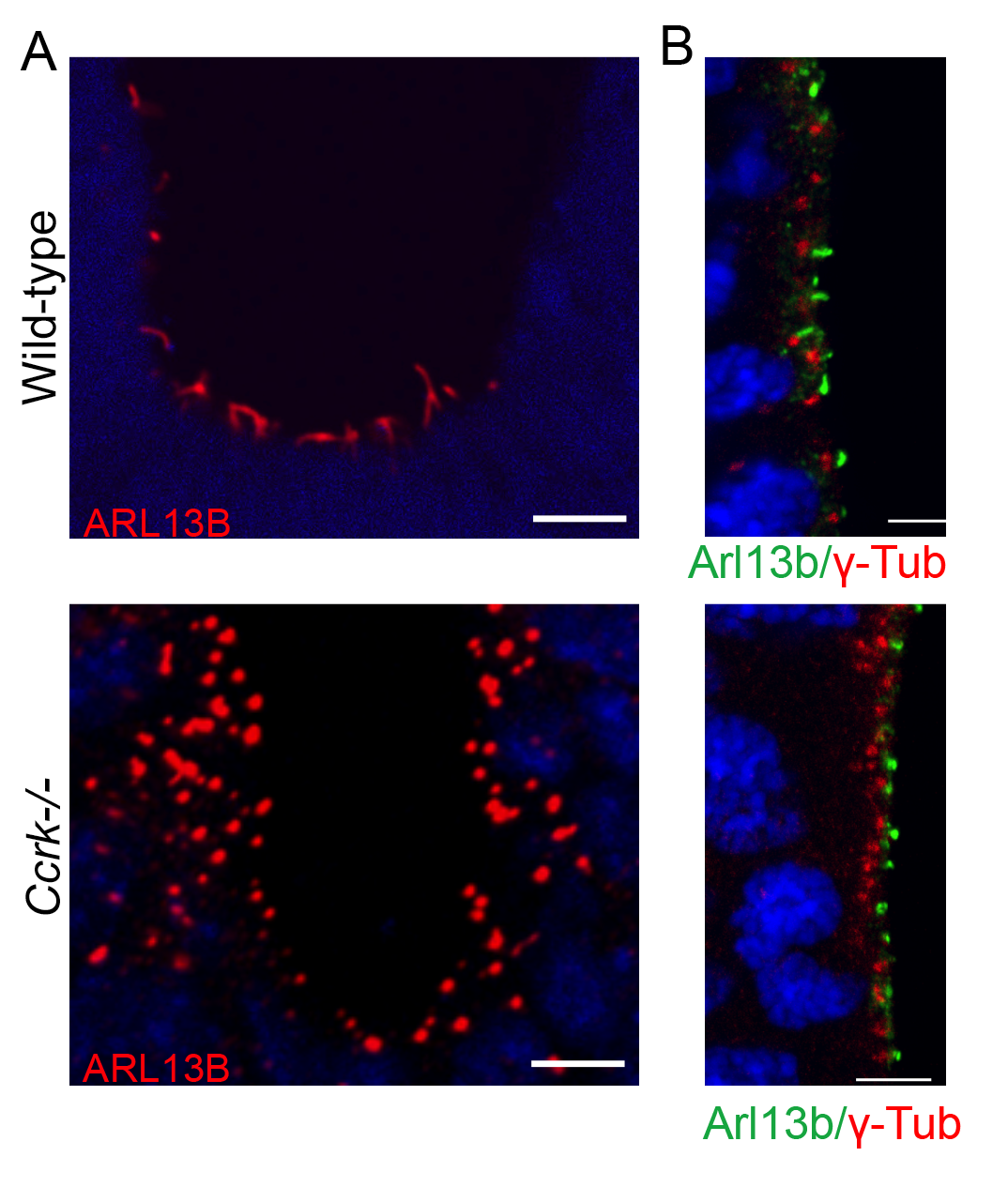

Supplement: S11 Fig — (A) Low magnification confocal images of the ventral neural tubes of E10.5 wild-type and Ccrk mutants stained for Arl13b (red) and DAPI (blue). Note that cilia were abundantly generated in the mutants. (B) High magnification images of neural tube cilia stained for γ-tubulin (red) to highlight basal bodies and Arl13b (green) to highlight cilia. Scale bars are 10 and 4 μm in A and B, respectively. (TIF) [file pgen.1006912.s011.tif]

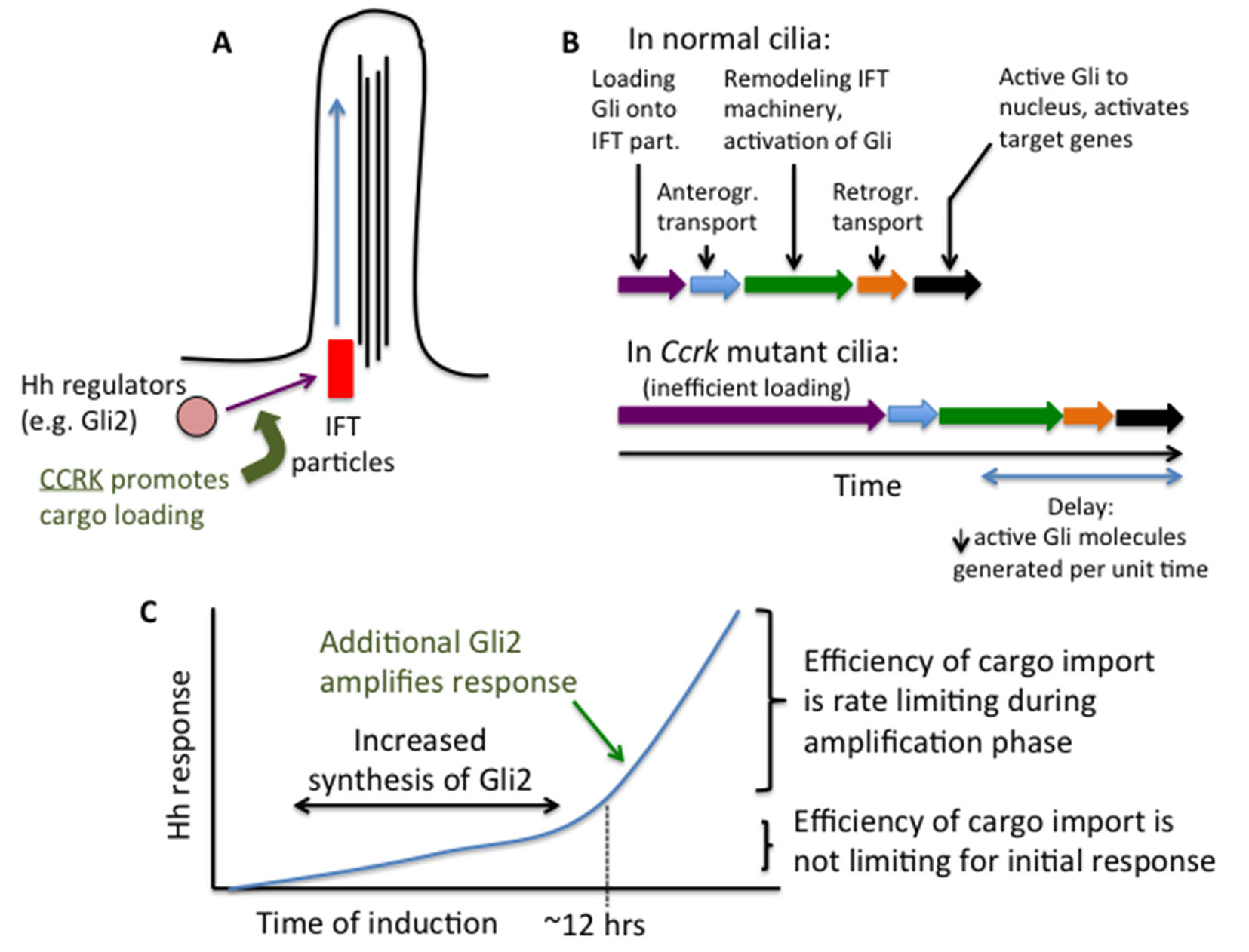

Supplement: S12 Fig — (A) We propose that CCRK acts through an unknown mechanism to promote the loading of ciliary cargo onto IFT particles so that they may be imported into the cilium. Such cargo includes regulators of Hh signal transduction (e.g. Gli2), tubulin dimers, and potentially, regulators of ciliary assembly/disassembly. (B) An individual Gli transcription factor molecule must first be loaded onto transport machinery and then transported towards the cilium tip where it can be activated (via its dissociation from Sufu). Once activated, the Gli molecule must be transported back to the cell body where it can enter the nucleus and activate target genes. We suggest that the initial step (loading and transport) is inefficient in Ccrk mutant cells. This would have the effect of delaying the time required for an individual molecule to be imported, activated, and function. Hence, fewer activated Gli molecules would be generated in the Ccrk mutant per unit of time. (C) During the period of Hh pathway induction, normal cells show two phases: an initial phase of slow increase, during which Gli2 is upregulated (due to feedback), and a second phase when the magnitude of the response is amplified (possibly due to the increased levels of Gli2). We suggest that the initial phase is relatively insensitive to the efficiency of ciliary import of Hh pathway regulators, but the transport efficiency is rate limiting for the response during the amplification phase. Thus, the decrease in the rate of ciliary transport of Hh pathway regulators in Ccrk mutant cells has very little effect during the initial phase, but it has a dramatic effect on the response during the amplification phase. (TIF) [file pgen.1006912.s012.tif]

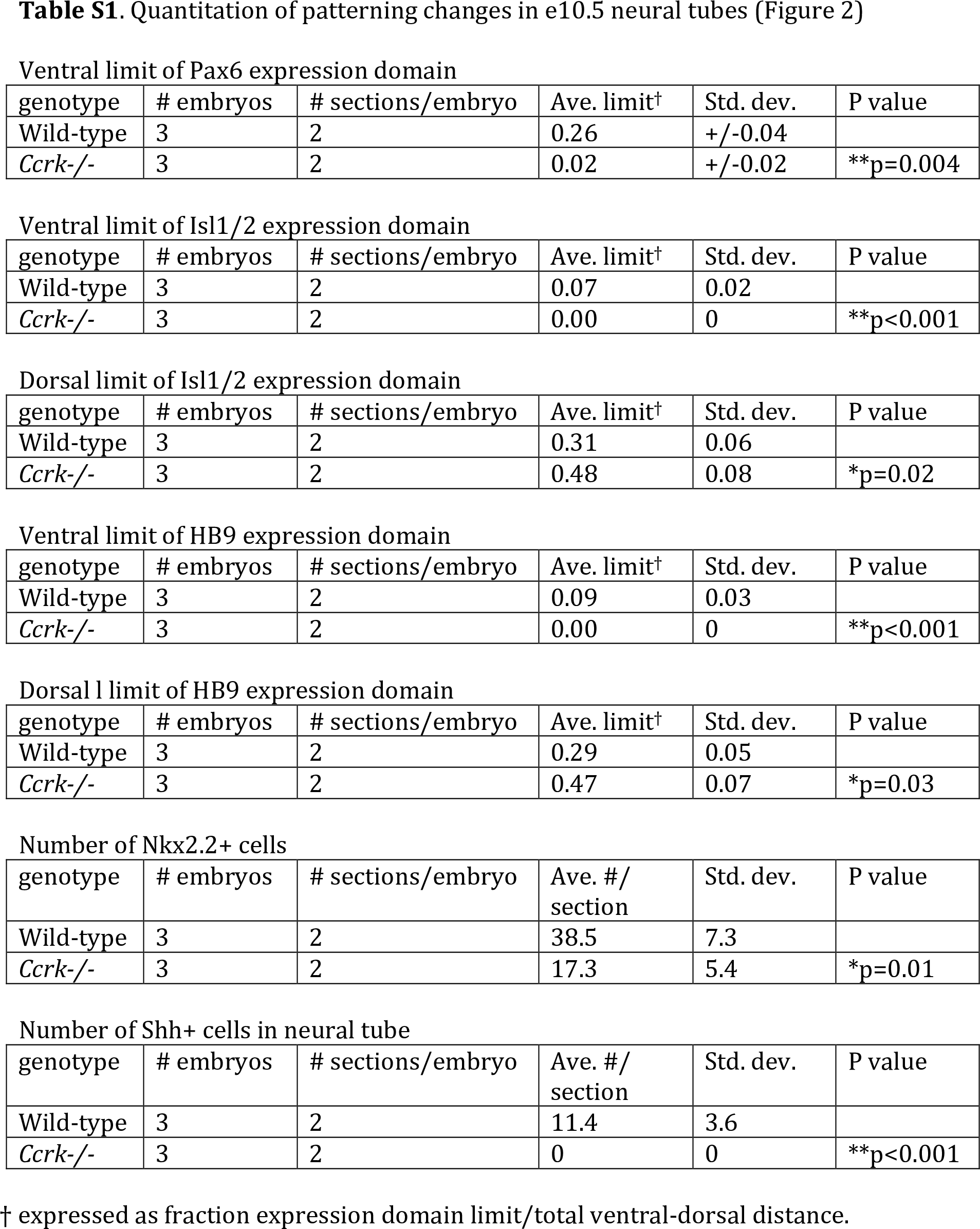

Supplement: S1 Table — Immunostained sections from embryos of Wild-type and Ccrk-/- genotypes were analyzed with respect to a) the position of dorsal and ventral limits of expression domains as a fraction of neural tube size (Pax6, Isl1/2, HB9) or b) numbers of marker positive cells per section (Nkx2.2 and Shh). Numbers of embryos per genotype and numbers of sections per embryo analyzed are shown, in addition to average values, standard deviations, and P values from Student’s t-tests. (TIF) [file pgen.1006912.s013.tif]

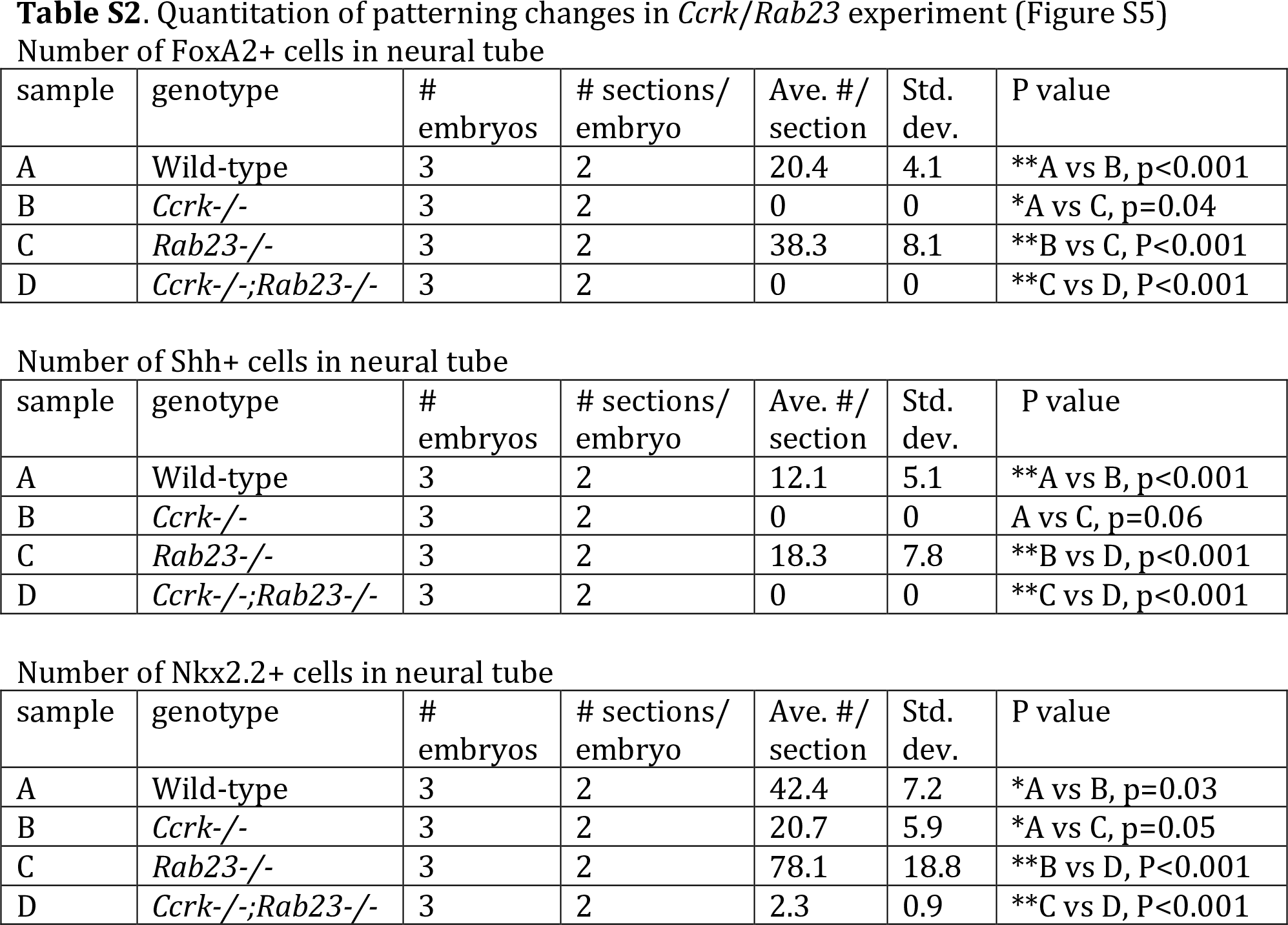

Supplement: S2 Table — Immunostained sections from embryos of Wild-type, Ccrk-/-, Rab23-/-, and Ccrk-/-Rab23-/- genotypes were analyzed with respect to numbers of FoxA2+, Shh+, and Nkx2.2+ cells per neural tube section. Numbers of embryos per genotype and numbers of sections per embryo analyzed are shown, in addition to average values, standard deviations, and P values from Student’s t-tests. (TIF) [file pgen.1006912.s014.tif]

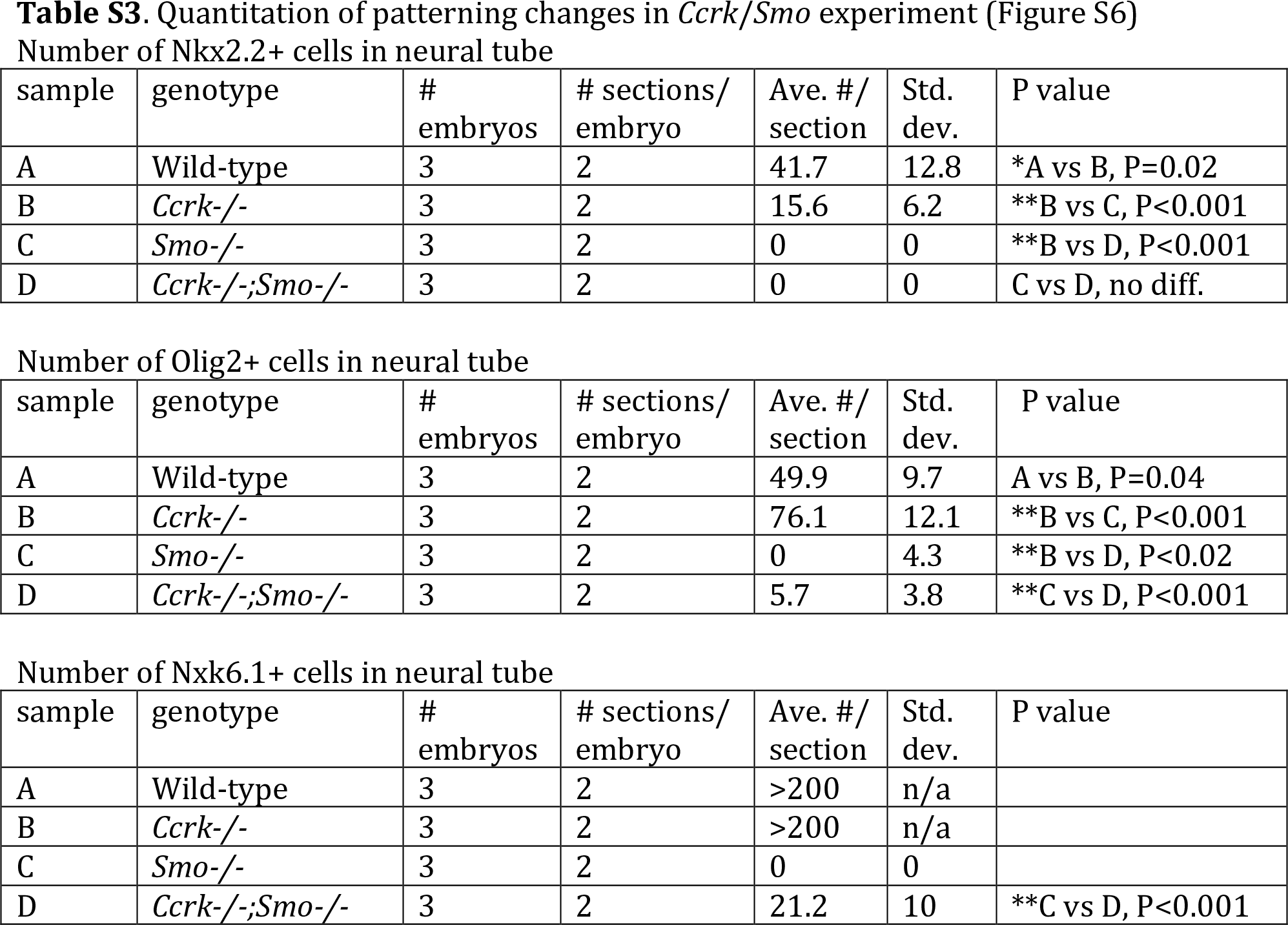

Supplement: S3 Table — Immunostained sections from embryos of Wild-type, Ccrk-/-, Smo-/-, and Ccrk-/-Smo-/- genotypes were analyzed with respect to numbers of Nkx2.2+, Olig2+, and Nkx6.1+ cells per neural tube section. Numbers of embryos per genotype and numbers of sections per embryo analyzed are shown, in addition to average values, standard deviations, and P values from Student’s t-tests. (TIF) [file pgen.1006912.s015.tif]

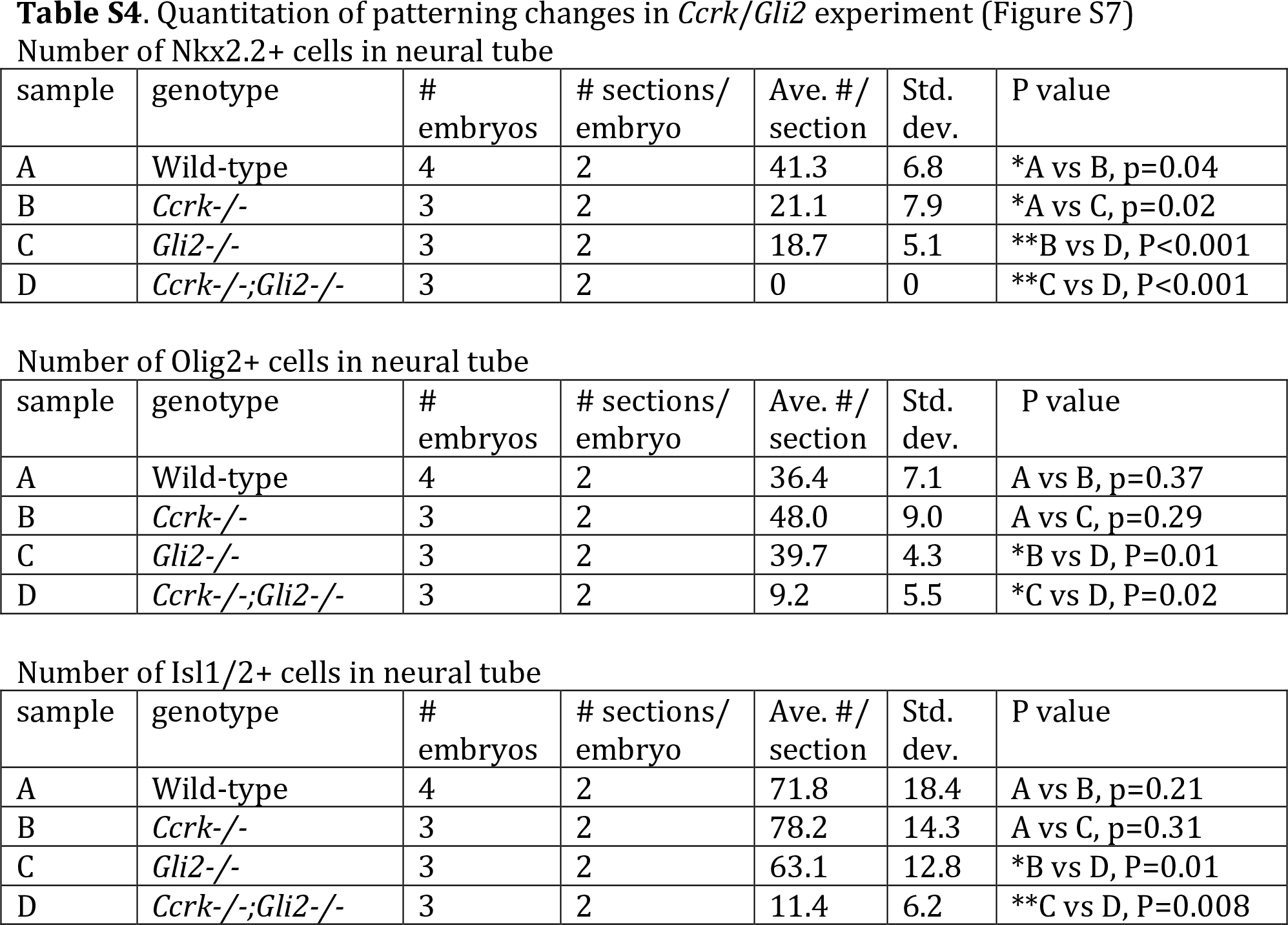

Supplement: S4 Table — Immunostained sections from embryos of Wild-type, Ccrk-/-, Gli2-/-, and Ccrk-/-Gli2-/- genotypes were analyzed with respect to numbers of Nkx2.2+, Olig2+, and Isl1/2+ cells per neural tube section. Numbers of embryos per genotype and numbers of sections per embryo analyzed are shown, in addition to average values, standard deviations, and P values from Student’s t-tests. (TIF) [file pgen.1006912.s016.tif]

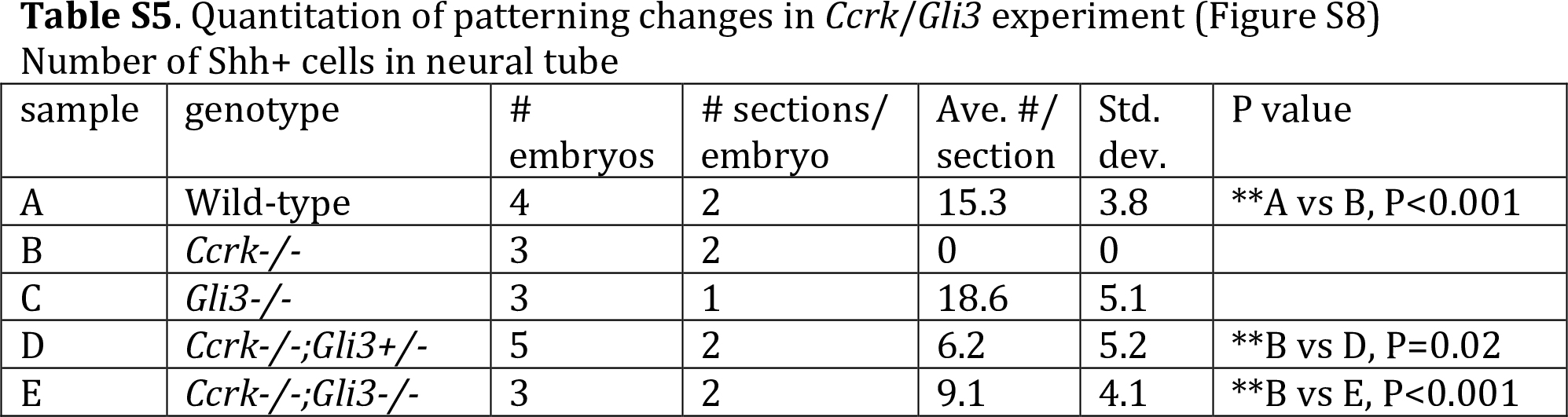

Supplement: S5 Table — Immunostained sections from embryos of Wild-type, Ccrk-/-, Gli3-/-, Ccrk-/-Gli3+/- and Ccrk-/-Gli3-/- genotypes were analyzed with respect to numbers of Shh+ cells per neural tube section. Numbers of embryos per genotype and numbers of sections per embryo analyzed are shown, in addition to average values, standard deviations, and P values from Student’s t-tests. (TIF) [file pgen.1006912.s017.tif]
